# Zinc finger nucleases for targeted mutagenesis and repair of the sickle-cell disease mutation: An *in-silico* study

**DOI:** 10.1186/1471-2326-12-5

**Published:** 2012-05-14

**Authors:** Misaki Wayengera

**Affiliations:** 1 Unit of Genetics, Genomics & Theoretical Biology, Dept of Pathology, School of Biomedical Science, College of Health Sciences, Makerere University, P o Box 7072, Kampala, Uganda

## Abstract

**Background:**

Sickle cell disease (or simply, SCD) is an inherited hemoglobinopathy which is mostly prevalent among persons of African descent. SCD results from a monogenic (Hemoglobin, beta) point-mutation (substitution of the base **A**denine with **T**hymine at position six) that leads to replacement of the amino acid glutamic acid (E) with valine (V). Management of SCD within resource-poor settings is largely syndromic, since the option of cure offered by bone-marrow transplantation (BMT) is risky and unaffordable by most affected individuals. Despite previous reports of repair and inhibition of the sickle beta-globin gene and messenger ribonucleic acids (mRNAs), respectively in erythrocyte precursor cells via gene-targeting using an oligomer-restriction enzyme construct and *either* ribozyme- *or* RNA-DNA chimeric oligonucleotides (or simply third strand binding), gene-therapy to treat SCD still remains largely preclinical. In the wake of the advances in target- gene- mutagenesis and repair wrought by zinc finger *nuclease* (ZFN) technology, it was hypothesized that SCD may be cured by the same. The goal of this study thus, was constructing a database of zinc finger arrays (ZFAs) and engineering ZFNs, that respectively bind and cleave within or around specific sequences in the sickle hemoglobin, beta (−*β*^S^) gene.

**Methods and results:**

***First*,** using the complete 1606 genomic DNA base pair (bp) sequences of the normal hemoglobin-beta (*β*^A^) chain gene, and the ZiFiT-CoDA-ZFA software preset at default, 57 three-finger arrays (ZFAs) that specifically bind 9 base-pair sequences within the normal hemoglobin-beta chain, were computationally assembled. ***Second*,** by serial linkage of these ZFAs to the Flavobacterium *okeanokoites* endonuclease Fok I― *four* ZFNs with unique specificity to >24 bp target-sequences at the genomic contextual positions 82, 1333, 1334, and 1413 of the β^A^ chain-gene were constructed *in-silico*. ***Third*,** localizing the point-mutation of SCD at genomic contextual position −69-**70**-71- bp (a position corresponding to the 6^th^ codon) of the *β*^A^ chain-gene, inspired the final design of *five* more ZFNs specific to >24 bp target-sequences within the 8,954 bp that are genomically adjacent to the 5′ end of the *β*^A^ chain-gene.

**Conclusions:**

This set of 57 ZFAs and 9 ZFNs offers us gene-therapeutic precursors for the targeted mutagenesis and repair of the SCD mutation or genotype.

## Background

Sickle cell diseases (SCD) or sickle cell anemia (SCA) is a hemoglobinopathy that is mostly common among persons of African descent [1]. SCD arises from a single, point-mutation (base-substitution of **A**denine with **T**hymine in the sixth codon: C**A**G → C**T**G) of the gene coding for the beta chain of the Hemoglobin molecule [2]. The phenotypic consequence of this substitution is a replacement of the amino acid glutamic acid (E) with valine (V) [1,2]. Homozygous expression of this mutant globin genotype (SS) causes SCD, while the heterozygous genotype (AS) is termed the “sickle cell trait” [1,2]. Unlike the case observed in most normal adult humans where the commonest hemoglobin type (hemoglobin A or Hb A) is a tetramer (which contains 4 subunit proteins- *α*_2_*β*_2_^A^ that are non-covalently bound together), patients with SCD have an adult hemoglobin type with two mutant *β* subunits (called *β*^S^) called hemoglobin S (or simply, Hb S) [3,4]. Hb S has a high predilection to crystallize under conditions of low oxygen-pressure such as may occur following physical or pathological-stress. Specifically, formation of intracellular S crystals causes polymerization of red blood cells, reduced oxygen uptake and or carriage, a as well as clogging of small blood vessels [5,6]. Overall, although the clinical syndrome of SCD is diversely-wide, its hallmark is a devastating group of *symptoms and signs* that are collectively known as a ‘sickle cell crisis’ [7,8]. About 200,000 new born babies within Africa recessively inherent the double autosomal-sickle cell genotype each year— a figure that constitutes approximately 66.6% of the children born with haemoglobinopathies worldwide [9]. Previously studies have shown that persons with heterozygosity for *β*^S^ (Hb SA) or the sickle cell trait are protected against infection by malaria causing protozoa [10]. This, together with findings of an equally high-incidence or common-distribution of the sickle cell trait within the malaria-belt, has led to the proposition that this trait emerged as an evolutionary adaptation of the human-host to infection with plasmodia [11]. The medical management of SCD remains an area of particular challenge [12]. Specifically, despite advances made towards curing SCD through bone-marrow transplantation [13,14], the resource-intensive nature of this approach has made it impossible for the most affected populations of Africa to access. Thus, care for affected individuals (homozygous SS) here still mostly revolves around syndromic management, with or without agents that increase fetal hemoglobin (Hb F) [15]. *Improvements are obviously sought here.*

By virtue of its monogenic, point-mutant origin, SCD has attracted several attempts for gene therapy. For instance, as early as 1991, Shesely ED, et *al.*[16] described a technique for the correction of a human *β*^S^ globin gene to the normal *β*^A^ allele by homologous recombination in the mouse-human hybrid cell line BSM using an oligomer-restriction enzyme construct. In 1998, Lans N, et *al.*[17,18] reported ribozyme mediated deletion and augmentation of the sickle-cell (β^S^) mutation with fetal haemoglobin levels in the red cells. Selective inhibition of beta-globin RNA transcripts by antisense RNA molecules has equally been tried as a strategy to reduce levels of Hb S polymerization in red blood cells and the symptoms associated with SCD [19-21]. Pace BS, et *al.*[21] specifically identified antisense RNA targets in the beta-globin gene other than the homologous regions in gamma-globin, proposing that gene therapy strategies which combine gamma-globin induction along with beta-globin inhibition using antisense vectors may yield more favorable anti-sickling effects longterm. Amosova O, et *al.*[22],on the other hand, reported third-strand directed repair of the sickle cell mutation using RNA-DNA chimeric oligonuceotides (COs) [23,24] achieved by shortening the psoralen linker to enhance the specificity of photoadduct formation at the desired mutant T residue site. Despite these notable advances, the place for gene-replacement or repair therapy in SCD has remained rather experimental [25], with no clinical trials of any of the above approaches in human populations yet reported.

Basing on the more-recent developments in targeted mutagenesis (genome-editing) and gene-repair wrought by zinc finger *nuclease* (ZFN) technology [26-32], it was hypothesized that the single, point mutation responsible for SCD can be abrogated using similar approaches. Specifically, Zinc finger nucleases-ZFNs, which are artificial, hybrid restriction enzymes created by covalently linking a DNA-binding zinc finger (Zif) domain (composed of three to six finger-arrays) to the non-specific DNA cleavage domain (or simply F_N_) of the Flavobacterium *okeanokoites* bacteria restriction endonuclease, FokI [33]; have recently become a powerful tool for primarily editing host genomes. Following induction of a double strand break (DSB) within the target DNA, repair occurs, either by homologous recombination (HR) or non-homologous end-joining (NHEJ) [26-32]. Perez et E *al*. [30]-using engineered ZFNs targeting human CCR5, previously demonstrated establishment of HIV-1 resistance in CD4+ T cells through generation of a doublestrand break (DSB) at predetermined sites in the CCR5 coding region upstream of the natural CCR5D32 mutation. Holmes N et *al*. [31] have demonstrated control of HIV-1 infection within NSG-mice transplanted with human hematopoietic stem/progenitor cells modified by zinc-finger nucleases targeting CCR5. Most recently, Wilen CB, et *al.*[32] successfully engineered HIV-Resistant Human CD4+ T Cells using CXCR4-Specific Zinc-Finger Nucleases (ZFN). This evidence, along with on-going improvements in the design and engineering of lentiviral [33,34] and parvovirus [35] vectors (LV and PV, respectively) for ex-*vivo* or in-*vivo* gene-delivery and transduction of erythroid precursors, suggests that the sickle cell mutation may be abrogated in erythroid bone marrow precursor with appropriate ZFNs. Indeed, we are aware that Sangamo Biosciences (http://www.sangamo.com/index.html)--one of the leading industries in ZFN-technology, has already focused its ZFN-mediated gene-editing technology to providing a unique solution for the treatment of monogenic diseases like hemophilia and SCA. Their ZFAs or ZFNs are, however, not publically available.

Thus, the specific goal of this study was to construct a database of zinc finger arrays (ZFAs) and engineer ZFNs that respectively specifically bind and cleave within or around the sickle hemoglobin beta (−β^S^) gene mutation.

## Methods

### Identification of HBB gene-specific ZFAs and ZFNs

No in-vivo or in-vitro experiments accompanied this bioinformatics study, and thereby no ethical approval and Consent was sought from the author’s institutional IRB.

#### Design

*In-silico* informatics

#### Materials and software

FASTA format of the nucleotide sequences of the entire Hemoglobin, beta-gene (provided in Additional file 1; the NCBI accession number provided at end) and the Zinc-Finger Nuclease-Consortium’s software CoDA-ZiFiT-ZFA and CoDA-ZiFiT-ZFN [36,37] (see software and availability section for URL link).

#### Interventions

The FASTA format of the nucleotide sequences of the hemoblobin, beta gene were separately fed into the user interfaces of CoDA-ZiFiT-ZFA and the CoDA-ZiFiT-ZFN, both of which were pre-set at default, with a spacer-option of 5–9 bp selected for the latter.

#### Measured variables

Lists of ZFAs and ZFNs, inclusive of graphic maps of their action in the genomic context of HB, beta, were generated as per the user protocol [36,37]. Another array of five ZFNs specific to >24 bp target-sequences within the adjacent 8,954 bp to the 5′ end of the *β*^A^ chain-gene; was also engineered.

### Software and database availability

The ZFN consortium CoDA-ZiFiT-ZFA/ZFN software and algorithms used are available at the following url: http://www.zincfingers.org/scientific-background.htm

The NCBI gene database hosting the HBB gene, is available at the following url: http://www.ncbi.nlm.nih.gov/gene/3043

## Results

### Zinc Finger Arrays (ZFAs) targeting hemoglobin, beta (β^A^) gene sequences

**Figure 1:**
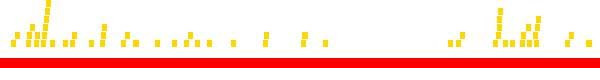
**Schematics of the ZFA- target binding sites within the hemoglobin, beta gene.** This figure graphically illustrates the binding sites of the *57* ZFAs that specifically target sequences within the genomic context of the hemoglobin, beta gene. Hits (blue, green, and gold bars) represent targets along the gene (red bars). ZFA hits in the graphic are color-coded based on spacer size (5 bp = Blue; 6 bp = Green; 7 bp = Gold).

*First*, using the 1,606 genomic nucleotide base pair (bp) sequences (inclusive of introns) encoding the normal hemoglobin-beta (β^A^) chain gene (*see*Additional file 1), and the ZiFiT-CoDA-ZFA [36,37] software preset at default, 57 three-finger arrays (ZFAs) that specifically bind 9 base-pair (bp) sequences within the normal hemoglobin-beta chain (*see*Additional file 2) were computationally assembled. Overall, there were ZFA binding within the first 3/5 and last 1/5 of the genomic contextual bp- sequences at 5′ and 3′ regions of the HB, beta gene (Figure 1). Zinc fingers (Zif or ZF)—such as those specific to the genomic nucleotide sequences of the HBB-gene (provided in Additional file 1), are protein motifs capable of targeted DNA-binding [26-32]. Each individual zinc finger usually recognizes three nucleotide bases, but many zinc fingers can be combined to generate an array capable, as in the case of our listed ZFAs of three fingers, of recognizing nine nucleotides [36,37]. Residues −1 to 6 (numbered relative to the start) of the alpha-helix of the ZFAs are responsible for the specific recognition of triplets of DNA sequences through the formation of base-specific contacts in the major groove of the double-stranded target DNA (see Additional file 2) [26-32,36,37]. Therefore, residues −1 to 6 within the ZFs’ alpha helixes are denoted as ‘recognition’ residues and are listed in N- to C-terminal direction; while all the other residues in the ZF are called the ‘backbone’ [36,37]. As a consequence, the recognition sequences of the ZFAs bind target DNA sites through amino acids −1 to 6 of the ‘recognition’ alpha helix in the *3'* to *5'* direction. The afore going reverse-pattern of target DNA recognition and binding can be confusing as the DNA target site is always referred to in the *5'* to *3'* direction, whereas amino acid sequences are referred to from the *N* to *C* terminus. In this section, ZFAs were assembled that are capable of cleaving at loci located within and adjacent to the 6^th^ or mutant codon of the sickle hemoglobin, beta (β**S**) gene, which is localized at genomic contextual positions of −69-**70**-71- bp (shown in colors, italics or bold in Additional file 1 and Additional file 2: one reverse or left ZFA alpha helix binding at position 51/41, and two forward or right ZFAs alpha helices binding at positions **70**/80 and 73/83; respectively). ZFAs can recombinantly be tagged to a non-specific nuclease *in-vivo*, a process that renders the non-specific nuclease specific to the binding sites of the ZFAs [38].

### Zinc Finger Nucleases (ZFN) targeting hemoglobin, beta (β^A^) gene sequences

**Figure 2:**
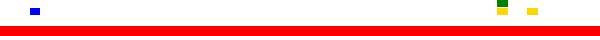
**Schematics of ZFN-cleavage sites within the hemoglobin, beta gene.** This figure graphically illustrates the cleavage sites of the *four* ZFNs targeting sequences within the human-genomic context of the hemoglobin, beta gene. Hits (blue, green, and gold bars) represent targets along the gene (red bars). ZFN hits in the graphic are color-coded based on spacer size (5 bp = Blue; 6 bp = Green; 7 bp = Gold).

*Second*, by serial linkage of these ZFAs to the Flavobacterium *okeanokoites* endonuclease Fok I of the type IIS class as Kim YG, et *al*. [38] previously achieved *in-vitro*; *four* ZFNs with unique specificity to >24 bp target-sequences at the genomic contextual positions 82, 1333, 1334, and 1413 of the β^A^ chain-gene were constructed *in-silico* (*see*Additional file 3 and Figure 2, respectively). Throughout the latter experiments, the description of the spacer regions was maintained at 5–7 base-pairs. Each specific ZFN has a left and right alpha-helical ‘recognition’ site bound to Fok I (*see* Table 1). Therefore, these ZFNs bind to their target >18 bp Hemoglobin, beta gene DNA sites as dimers, with each monomer using its zinc finger domain to recognize a ‘half-site’ of the targeted DNA sequence. *In-vivo*, dimerization of ZFNs is mediated by the FokI cleavage domain through cleavage of a five or six base pair ‘spacer’ sequence that separates the two inverted target ‘half sites’ [26-32]. Importantly, since the DNA-binding specificities of zinc finger domains can be re-engineered using various methods, customized ZFNs can, in principle, be constructed to specifically target almost any gene sequence [37]. A list of three ZFNs, inclusive of their −1 to 6 alpha-helical nucleotide binding domains (F1, F2, F3/F3, F2, F1) alongside the respective site specific sequence within the genomic context of the Hemoglobin, beta gene, are presented in Table 1. Graphic-analysis of the cleavage-pattern within the HB, beta gene induced by the *four* identified ZFNs revealed *one* of them to cleave at the 5′ end (position 82/106) while the other *three* cleaved at the 3′ end (positions 1,333/1,359, 1,334/1,359, and 1,413/1,439, respectively) (see, Figure 2).

**Table 1:** **ZFN targeting and cleaving within the hemoglobin, beta (*β***^**A**^**)-gene**

**Zinc Finger Nuclease (ZFN)**	**Left Fn; triplet- α-Helix**	**Right Fn; triplet- α-Helix**
-**targeting HBB (*β***^**A**^**)-DNA at 5′ Sites**		
**ZFN-unknown-SP-5-1**		
5**′_**82 cCGTTACTGCCCTGT**GGGGCAAGG**t 106_**′3**	F1; RNITLVR; (**ACG**)	F1; RNEHLKV; (**AGG**)
5**′_**82 g**GCAATGACG**GGACACCCCGTTCCa 106_**′3**	F2; QRSSLVR; (**GTA**)	F2; QSTTLKR; (**GCA**)
	F3; QDNTLRR; (**GCA**)	F3; RTEHLAR; (**GGG**)
-**targeting HBB (*β***^**A**^**)-DNA at 3′ Sites**		
**ZFN-unknown-SP-7-1**		
5**′_**1333 cTTCCTCCCACAGCTCC**TGGGCAACG**t 1359_**′3**	F1; QASNLLR; (**GAA**)	F1; RSQTLAQ; (**ACG**)
5**′_**1333 g**AAGGAGGGT**GTCGAGGACCCGTTGCa 1359_**′3**	F2; RQDNLGR; (**GAG**)	F2; QSTTLKR; (**GCA**)
	F3; RMDHLAG; (**TGG**)	F3; RSDHLSL; (**TGG**)
**ZFN-unknown-SP-6-1**		
5**′_**1334 tTCCTCCCACAGCTCC**TGGGCAACG**t 1359_**′3**	F1; RTDRLIR; (**GGA**)	F1; RSQTLAQ; (**ACG**)
5**′_**1334 a**AGGAGGGTG**TCGAGGACCCGTTGCa 1359_**′3**	F2; QSAHLKR; (**GGA**)	F2; QSTTLKR; (**GCA**)
	F3; RNTALQH; (**GTG**)	F3; RSDHLSL; (**TGG**)

### Zinc Finger Nucleases (ZFN) targeting within the adjacent 8,954 bp to the 5′ end of the β^A^ chain-gene

**Figure 3:**
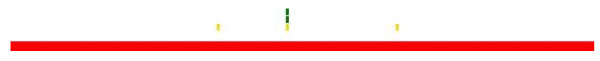
**Schematics of ZFN-cleavage sites within the adjacent 8,954 bp to the 5′ end of the hemoglobin, beta gene.** This figure graphically illustrates the cleavage sites of *five* ZFNs targeting sequences within 8,954 bp located to the 5′ end of the *β*^A^ chain gene. Two of the ZFNs cleaving closest to the 5′ end of the *β*^A^ chain-gene are shown in Table 2. Hits (blue, green, and gold bars) represent targets along the gene (red bars). ZFN hits in the graphic are color-coded based on spacer size (5 bp = Blue; 6 bp = Green; 7 bp = Gold).

*Third*, by localizing the point-mutation of SCD at genomic contextual position −69-**70**-71- bp of the β^A^ chain-gene sequences used (that is, within 6^th^ codon of the gene: *shown by green italicized letters* in Additional file 1), design of another array of five ZFNs specific to >18 bp target-sequences within the adjacent 8,954 bp to the 5′ end of the *β*^A^ chain-gene (*see*Additional file 4 for 8,954 bp to the 5′ end of the *β*^A^ chain-gene; and Additional file 5 for ZFN cleaving here) was inspired. Two of the ZFN cleaving closest to the 5′ end of the *β*^A^ chain-gene are shown in Table 2. A graphic summary of the distribution of cleavage patterns therein is shown in Figure 3.

**Table 2:** **ZFN targeting and cleaving within the adjacent 8,954 bp to the 5′ end of the hemoglobin, beta (*β***^**A**^**)-gene**

**Zinc Finger Nuclease (ZFN)**	**Left Fn; triplet- α-Helix**	**Right Fn; triplet- α-Helix**
**ZFN-unknown-SP-6-1**		
5**′_**4231 aGCCACCACCTTCT GA**TAGGCAGCC**t 4256_**′3**	F1; VPSKLKR; (**GGC**)	F1; DPSTLRR; (**GCC**)
5**′_**4231 t**CGGTGGTGG**AAGA CTATCCGTCGGa 4256_**′3**	F2; EAHHLSR; (**GGT**)	F2; QSTTLKR; (**GCA**)
	F3; IRHHLKR; (**GGT**)	F3; RRDGLAG; (**TAG**)
**ZFN-unknown-SP-6-2**		
5**′_**4234 cACCACCTTCTGAT AG**GCAGCCTGC**a 4259_**′3**	F1; MKHHLAR; (**GGT**)	F1; RGRNLEM; (**TGC**)
5**′_**4234 g**TGGTGGAAG**A CTATCCGTCGGACGt 4259_**′3**	F2; EAHHLSR; (**GGT**)	F2; DSSVLRR; (**GCC**)
	F3; QDGNLTR; (**GAA**)	F3; QGGTLRR; (**GCA**)

## Discussion and conclusions

I present here a novel set of *57* zinc finger *arrays* (ZFAs) and 9 zinc finger *nucleases* (ZFNs) ― that constitute gene-therapeutic precursors for the targeted mutagenesis and repair of the SCD mutation or genotype. Specifically, although SCD results from a monogenic (Hemoglobin, beta) point-mutation (substitution of A with T) that has attracted extensive interest for target gene-therapy [16-25], the place for gene-replacement or repair therapy in SCD has remained rather experimental [25], and no clinical trials of any of the above approaches in human populations are yet to be reported. Basing on the more-recent developments in targeted mutagenesis (genome-editing) and gene-repair wrought by zinc finger *nuclease* (ZFN) technology [26-32], it was hypothesized that the single, point mutation responsible for SCD can be abrogated using similar approaches. To this end, we sought to construct a database of zinc finger arrays (ZFAs) and engineer ZFNs that specifically bind and cleave within or around the sickle hemoglobin, beta (−*β*^S^) gene. Now, we present a database of *57* ZFAs (see, Additional file 2 and Figure 1) specific to the *β*^A^ gene (*see,*Additional file 1 for genomic contextual sequences). Using these ZFAs, we also constructed *four* ZFNs (shown in Additional file 3 and Figure 2) cleaving specifically within the same *β*^A^ gene. *Three* of these *four* ZFN are shown in Table 1. Because the point-mutation of SCD is located at genomic contextual position −69-**70**-71- bp of the *β*^A^ chain-gene sequences used (that is, within the 6^th^ codon of the gene), we were equally inspired to design another array of *five* ZFNs (shown in Additional file 5) specific to >24 bp target-sequences within the adjacent 8,954 bp to the 5′ end of the *β*^A^ chain-gene (*see*Additional file 4 for the 8,954 bp localized to the 5′ end of the *β*^A^ chain-gene). Two of the ZFN cleaving within these- 8,954 bp but closest to the 5′ end of the *β*^A^ chain-gene, are shown in Table 2, while Figure 3 offers a graphic summary of their distribution.

The above ZFAs and ZFNs may be applied towards the target mutagenesis or repair of the sickle cell mutation, in various ways. *Firstly*, splicing out the entire genomic region located between positions 5′-82/106*, and* 3′ *-*1,333/1,359 *or −*1,334/1,359 of the *β*^S^ globin gene may be achieved using the ZFNs shown in Table 1. This alone― when followed by the process of non-homologous end-joining (NHEJ) [26-32] of the residual double strand DNA break (DSB)’s edges could lead to deletion of over 80% bp-sequences of the defective *β*^S^ globin gene. Depending on the efficiency of the gene-delivery and transduction achieved by the vectors [34,35] used within erythroid precursors, *therefore*, this strategy alone offers the possibility of reducing the intracellular (*either* mature red blood cell, RBC *or* erythroid precursor) expression of the *β*^S^ globin gene, and ultimately curtailing the symptomatology resulting from S polymerization. Note that, because this approach does not involve deletion of the SCD point-mutation which is located at genomic contextual position −69-**70**-71- bp of the *β*^A^ chain-gene sequences used (that is, within the 6^th^ codon of the gene), it is only a means to functionally irreversibly inactivate the *β*^S^ globin gene. Thus, as a *second option*, another may purpose to delete the 5′ region before position −82/106 of the *β*^S^ globin gene, which contains the point-mutation of SCD. This can be achieved using the ZFN in Table 1 that cleaves at position 82/106 and any of the two ZFNs in Table 2 that cleave within the 8,954 bp localized to the 5′ end of the *β*^A^ chain-gene. Alternatively, however, secondary therapeutic events, including say (i) *either* repair of the target β^S^ globin gene cleavage-site by *either* recruiting the homologous recombination (HR*)* pathway through providing a normal, non-mutant *β* globin gene template for repair of the spliced ‘mutant (disease causing)’ region [26-32,36,37] (ii) or supplementary replacement of this pathogenic-spliceon with a corresponding 5′ exon of γ-globin in a similar way as Lans N, et *al.*[17] and Weatherall DJ. [18] did with a 3′ exon, may be sought. Pace BS, et *al.*[21] has proposed that gene therapy strategies which combine gamma-globin induction along with beta-globin inhibition may yield more favorable anti-sickling effects in the longterm. *Lastly*, perhaps completely novel gene-therapeutic strategies devised in future may equally explore the target-DNA-binding mechanisms inherent in our ZFAs or ZFN to repair the mutant codon of the *β*^S^ globin gene.

**Figure 4:**
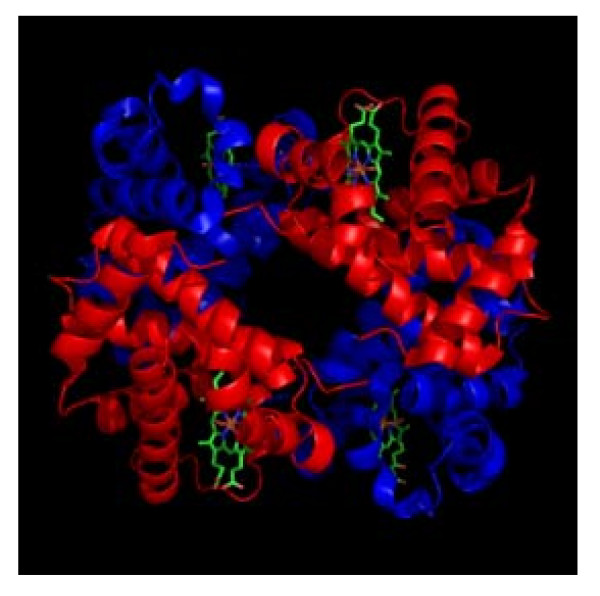
**Crystal Structure of adult hemogblonin, A.** This figure details the crystal structure of adult hemogblonin, A as deposited by Silva MM, Rogers PH, & Arnone A [43].

Our study presents a number of limitations that need to be taken into account. *First*, it is vital to note that the work is limited to only sequence analyses and accompanied by no *in-vitro* proof of concept studies. This should be attributed to the apparent limited resource-capacity in our Lab, although the same may be ratified by the fact that these very methods [36,37] have previously been used to successfully assemble ZFAs and engineer ZFNs that are experimentally safe and effective [26-32]. That said, it is imperative that further optimization be incorporated, including say: (i) modular analysis and assembly [39] to add on one, two or even three other ZFA onto our currently 3 finger-arrays so as to enhance specificity and avoid off-target host-genome toxicity; and (ii) *in-vivo* assembly and testing for efficacy, say by using either a bacteria-one hybrid (B1H) or yeast one-hybrid (Y1B) system to further inform the best ZFNs to use [40]. Modifications to the cleavage domain in order to generate a hybrid capable of functionally interrogating the ZFN dimer interface so as to prevent homodimerization, while-still enhancing the efficiency of cleavage [41], is also possible. *Second,* additional pre-clinical experiments employing *either* humanized-mouse cell models [16] or erythroid precursors cells, are still required to assess the efficacy and safety of these ZFNs and their transducing vectors [34,35,42]. Perhaps, those experiments that Wilen CB, et *al.*[32] recently conducted to assess the safety and efficacy of the Ad5/F35 vector carrying CXCR4-Specific Zinc-Finger Nucleases they used to engineer HIV-resistant human CD4+ T cells, may suffice. Specifically, it is important to (i) compare genome profiles and the hemoglobin, beta gene- sequence-profiles of the erythroid precursors targeted, and (ii) evaluate biophysical profiles of the mutagenic or repair resultant, presumably adult hemoblogin (HB*β*^S→A^) produced, including analyzing its crystal structure (see Figure 4 for crystal structure of the normal adult hemoglobin A) [43-45]*Third,* its reasonable for one to question how the lentiviral or parvovirus vectors carrying and transducing a diploid (or pair) copy of these ZFN will be used in the clinics within resource limited settings where SCD is most prevalent [25,46]. Our translational projection or proposition is to have this attempted via *in-vivo* gene-delivery and transduction, rather than the expensive *ex-vivo* manipulation. Therefore, sub-dermal, intravenous or intra-osseous routes of injection or infusion for *in-vivo* use of these vectors as a less-Labour-intensive and affordable gene-delivery and transduction alternative to *ex-vivo* manipulation must seriously be considered and tried despite the evidence of low efficiency of gene-delivery and transduction offered by the *in-vivo* routes, when compared to *ex-vivo* gene delivery [34,35]. *Fourth*, zinc finger nucleases targeting host-genes have recently been found to cleave off-target loci [47], and these off-targets-though minimized by reducing the binding energy of ZFN, could not be predicted by in-silico methods [48].

In conclusion, this set of 57 zinc finger arrays (ZFAs) and 9 zinc finger nucleases (ZFNs) ― offers us gene-therapeutic precursors for the targeted mutagenesis and repair of the SCD mutation or genotype. Specifically, the same may be used to either *functionally* or *structurally* abrogate the *β*^S^ globin gene. Alternatively, supplementary replacement of this pathogenic-spliceon with a corresponding 5′ exon of γ-globin may be possible. Lastly, novel gene-therapeutic strategies devised in future may equally explore the target-DNA binding and cleaving mechanisms inherent in our ZFAs or ZFN to replace or repair the mutant codon of the *β*^S^ globin gene.

## Competing interests

There are no competing interests to declare.

## Authors’ contributions

WM conceived the idea for this article, designed and undertook the experiments, and wrote the MS.

## Accession numbers

The NCBI gene identity of the Hemoglobin, beta gene is |3043|; whereas the NCBI reference for the 8.95 kb region from base 5190000 to 5198951 is |NT_009237.18|

## Pre-publication history

The pre-publication history for this paper can be accessed here:

http://www.biomedcentral.com/1471-2326/12/5/prepub

## Supplementary Material

Additional file 1 **A detailed list of the 1,606 nucleotide bases within the Hemoglobin, beta gene.** This file offers FASTA-format listing of the 1,606 nucleotide bases within the Hemoglobin, beta gene (gene identity |3043|). Click here for file

Additional file 2 **A detailed list of the*57*ZFAs specific to the Hemoglobin, beta gene-sequences.** This file lists the *57* ZFAs that specifically bind sequences within the Hemoglobin, beta gene Click here for file

Additional file 3 **A detailed list of the*four*ZFNs specific to the Hemoglobin, beta gene-sequences.** This file lists the *four* ZFNs that specifically bind and cleave sequences within the Hemoglobin, beta gene Click here for file

Additional file 4 **A detailed list of the 8,954 bp located to the 5′ end of the*β***^**A**^**chain gene.** This file offers FASTA-format listing of the 8,954 bp located to the 5′ end of the *β*^A^ chain gene. (NCBI reference sequence 5190000 to 5198951 is |NT_009237.18|) Click here for file

Additional file 5 **A detailed list of the*five*ZFNs specific to the 8,954 bp located to the 5′ end of the*β***^**A**^**chain gene.** This file lists the *fives* ZFNs that specifically bind and cleave sequences within the 8,954 bp located to the 5′ end of the *β*^A^ chain gene. Click here for file
