# Supplementary material for: Zinc finger nucleases for targeted mutagenesis and repair of the sickle-cell disease mutation: An in-silico study
Source: BMC Blood Disord. 2012 May 14;12:5. doi: 10.1186/1471-2326-12-5 (PMC3407482; doi:10.1186/1471-2326-12-5)
Supplement: Additional file 1 — A detailed list of the 1,606 nucleotide bases within the Hemoglobin, beta gene. This file offers FASTA-format listing of the 1,606 nucleotide bases within the Hemoglobin, beta gene (gene identity |3043|). [file 1471-2326-12-5-S1.doc]

NCBI Reference Sequence: NG_000007.3; Gene ID: 3043,


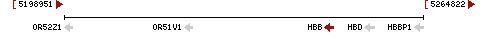


>gi|28380636:70545-72150 Homo sapiens beta globin region (HBB@); and hemoglobin, beta (HBB); and hemoglobin, delta (HBD); and hemoglobin, epsilon 1 (HBE1); and hemoglobin, gamma A (HBG1); and hemoglobin, gamma G (HBG2), RefSeqGene on chromosome 11

ACATTTGCTTCTGACACAACTGTGTTCACTAGCAACCTCAAACAGACACCATG*GTG***CAT***CTG*ACT**CCT*GA***

***G***GAG**AAG***TCT*GCCGTTACTGCCCTGTGGGGCAAGGTGAACGTGGATGAAGTTGGTGGTGAGGCCCTGGGC

AGGTTGGTATCAAGGTTACAAGACAGGTTTAAGGAGACCAATAGAAACTGGGCATGTGGAGACAGAGAAG

ACTCTTGGGTTTCTGATAGGCACTGACTCTCTCTGCCTATTGGTCTATTTTCCCACCCTTAGGCTGCTGG

TGGTCTACCCTTGGACCCAGAGGTTCTTTGAGTCCTTTGGGGATCTGTCCACTCCTGATGCTGTTATGGG

CAACCCTAAGGTGAAGGCTCATGGCAAGAAAGTGCTCGGTGCCTTTAGTGATGGCCTGGCTCACCTGGAC

AACCTCAAGGGCACCTTTGCCACACTGAGTGAGCTGCACTGTGACAAGCTGCACGTGGATCCTGAGAACT

TCAGGGTGAGTCTATGGGACGCTTGATGTTTTCTTTCCCCTTCTTTTCTATGGTTAAGTTCATGTCATAG

GAAGGGGATAAGTAACAGGGTACAGTTTAGAATGGGAAACAGACGAATGATTGCATCAGTGTGGAAGTCT

CAGGATCGTTTTAGTTTCTTTTATTTGCTGTTCATAACAATTGTTTTCTTTTGTTTAATTCTTGCTTTCT

TTTTTTTTCTTCTCCGCAATTTTTACTATTATACTTAATGCCTTAACATTGTGTATAACAAAAGGAAATA

TCTCTGAGATACATTAAGTAACTTAAAAAAAAACTTTACACAGTCTGCCTAGTACATTACTATTTGGAAT

ATATGTGTGCTTATTTGCATATTCATAATCTCCCTACTTTATTTTCTTTTATTTTTAATTGATACATAAT

CATTATACATATTTATGGGTTAAAGTGTAATGTTTTAATATGTGTACACATATTGACCAAATCAGGGTAA

TTTTGCATTTGTAATTTTAAAAAATGCTTTCTTCTTTTAATATACTTTTTTGTTTATCTTATTTCTAATA

CTTTCCCTAATCTCTTTCTTTCAGGGCAATAATGATACAATGTATCATGCCTCTTTGCACCATTCTAAAG

AATAACAGTGATAATTTCTGGGTTAAGGCAATAGCAATATCTCTGCATATAAATATTTCTGCATATAAAT

TGTAACTGATGTAAGAGGTTTCATATTGCTAATAGCAGCTACAATCCAGCTACCATTCTGCTTTTATTTT

ATGGTTGGGATAAGGCTGGATTATTCTGAGTCCAAGCTAGGCCCTTTTGCTAATCATGTTCATACCTCTT

ATCTTCCTCCCACAGCTCCTGGGCAACGTGCTGGTCTGTGTGCTGGCCCATCACTTTGGCAAAGAATTCA

CCCCACCAGTGCAGGCTGCCTATCAGAAAGTGGTGGCTGGTGTGGCTAATGCCCTGGCCCACAAGTATCA

CTAAGCTCGCTTTCTTGCTGTCCAATTTCTATTAAAGGTTCCTTTGTTCCCTAAGTCCAACTACTAAACT

GGGGGATATTATGAAGGGCCTTGAGCATCTGGATTCTGCCTAATAAAAAACATTTATTTTCATTGC

mRNA Transcript NM_000518.4

>gi|28302128|ref|NM_000518.4| Homo sapiens hemoglobin, beta (HBB), mRNA

ACATTTGCTTCTGACACAACTGTGTTCACTAGCAACCTCAAACAGACACCATGGTG*CAT***CTG**ACT*CCT***GA**

**GGAG***AAG*TCTGCCGTTACTGCCCTGT**GGGGCAAGG**TGAACGTGGATGAAGTTGGTGGTGAGGCCCTGGGC

AGGCTGCTGGTGGTCTACCCTTGGACCCAGAGGTTCTTTGAGTCCTTTGGGGATCTGTCCACTCCTGATG

CTGTTATGGGCAACCCTAAGGTGAAGGCTCATGGCAAGAAAGTGCTCGGTGCCTTTAGTGATGGCCTGGC

TCACCTGGACAACCTCAAGGGCACCTTTGCCACACTGAGTGAGCTGCACTGTGACAAGCTGCACGTGGAT

CCTGAGAACTTCAGGCTCCTGGGCAACGTGCTGGTCTGTGTGCTGGCCCATCACTTTGGCAAAGAATTCA

CCCCACCAGTGCAGGCTGCCTATCAGAAAGTGGTGGCTGGTGTGGCTAATGCCCTGGCCCACAAGTATCA

CTAAGCTCGCTTTCTTGCTGTCCAATTTCTATTAAAGGTTCCTTTGTTCCCTAAGTCCAACTACTAAACT

GGGGGATATTATGAAGGGCCTTGAGCATCTGGATTCTGCCTAATAAAAAACATTTATTTTCATTGC

**Key:** The bald **CTG** codon encodes leucine at position 3, three codonds downstream (position 6) of which lies the codon GAG (colored green) that encodes Glutamine in normal hemoglobin. The codon of glutamine (GAG) is replaced by GTG (coding valine) by a single substitution of A with T.
