# Supplementary material for: Zinc finger nucleases for targeted mutagenesis and repair of the sickle-cell disease mutation: An in-silico study
Source: BMC Blood Disord. 2012 May 14;12:5. doi: 10.1186/1471-2326-12-5 (PMC3407482; doi:10.1186/1471-2326-12-5)
Supplement: Additional file 2 — A detailed list of the57ZFAs specific to the Hemoglobin, beta gene-sequences. This file lists the 57 ZFAs that specifically bind sequences within the Hemoglobin, beta gene [file 1471-2326-12-5-S2.doc]

**Zinc Finger Site Type:** Array
**Zinc Finger Engineering Method:** CoDA
**Sequence Name** :
**Sequence Length**:1608
**Nucleotide Sequence** :nACATTTGCTTCTGACACAACTGTGTTCACTAGCAACCTCAAACAGACACCATGGTGCATCTGACTCCTGAGGAGAAGTCTGCCGTTACTGCCCTGTGGGGCAAGGTGAACGTGGATGAAGTTGGTGGTGAGGCCCTGGGCAGGTTGGTATCAAGGTTACAAGACAGGTTTAAGGAGACCAATAGAAACTGGGCATGTGGAGACAGAGAAGACTCTTGGGTTTCTGATAGGCACTGACTCTCTCTGCCTATTGGTCTATTTTCCCACCCTTAGGCTGCTGGTGGTCTACCCTTGGACCCAGAGGTTCTTTGAGTCCTTTGGGGATCTGTCCACTCCTGATGCTGTTATGGGCAACCCTAAGGTGAAGGCTCATGGCAAGAAAGTGCTCGGTGCCTTTAGTGATGGCCTGGCTCACCTGGACAACCTCAAGGGCACCTTTGCCACACTGAGTGAGCTGCACTGTGACAAGCTGCACGTGGATCCTGAGAACTTCAGGGTGAGTCTATGGGACGCTTGATGTTTTCTTTCCCCTTCTTTTCTATGGTTAAGTTCATGTCATAGGAAGGGGATAAGTAACAGGGTACAGTTTAGAATGGGAAACAGACGAATGATTGCATCAGTGTGGAAGTCTCAGGATCGTTTTAGTTTCTTTTATTTGCTGTTCATAACAATTGTTTTCTTTTGTTTAATTCTTGCTTTCTTTTTTTTTCTTCTCCGCAATTTTTACTATTATACTTAATGCCTTAACATTGTGTATAACAAAAGGAAATATCTCTGAGATACATTAAGTAACTTAAAAAAAAACTTTACACAGTCTGCCTAGTACATTACTATTTGGAATATATGTGTGCTTATTTGCATATTCATAATCTCCCTACTTTATTTTCTTTTATTTTTAATTGATACATAATCATTATACATATTTATGGGTTAAAGTGTAATGTTTTAATATGTGTACACATATTGACCAAATCAGGGTAATTTTGCATTTGTAATTTTAAAAAATGCTTTCTTCTTTTAATATACTTTTTTGTTTATCTTATTTCTAATACTTTCCCTAATCTCTTTCTTTCAGGGCAATAATGATACAATGTATCATGCCTCTTTGCACCATTCTAAAGAATAACAGTGATAATTTCTGGGTTAAGGCAATAGCAATATCTCTGCATATAAATATTTCTGCATATAAATTGTAACTGATGTAAGAGGTTTCATATTGCTAATAGCAGCTACAATCCAGCTACCATTCTGCTTTTATTTTATGGTTGGGATAAGGCTGGATTATTCTGAGTCCAAGCTAGGCCCTTTTGCTAATCATGTTCATACCTCTTATCTTCCTCCCACAGCTCCTGGGCAACGTGCTGGTCTGTGTGCTGGCCCATCACTTTGGCAAAGAATTCACCCCACCAGTGCAGGCTGCCTATCAGAAAGTGGTGGCTGGTGTGGCTAATGCCCTGGCCCACAAGTATCACTAAGCTCGCTTTCTTGCTGTCCAATTTCTATTAAAGGTTCCTTTGTTCCCTAAGTCCAACTACTAAACTGGGGGATATTATGAAGGGCCTTGAGCATCTGGATTCTGCCTAATAAAAAACATTTATTTTCATTGCn
**Selected Module Sets:**
**Selected Module Count:** 3
**Ignore Asp Overlap:**True

The results below are zinc finger arrays that can be constructed using CoDA. Note that other methods (including modular assembly and OPEN) can also potentially be used to target the input sequence of interest.”

**Sort By:**

**[

](http://zifit.partners.org/ZiFiT/CoDAZiFiTArray.aspx#ctl00_ContentPlaceHolder1_tree12_SkipLink)**

| [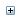](javascript:TreeView_ToggleNode(ctl00_ContentPlaceHolder1_tree12_Data,0,document.getElementById('ctl00_ContentPlaceHolder1_tree12n0'),'%20',document.getElementById('ctl00_ContentPlaceHolder1_tree12n0Nodes'))) | ZFA-unknown-1 40 t[GAGGTTGCT](http://bindr.gdcb.iastate.edu:8080/ZiFDB/controller/searchArray?site=GCTGTTGAG)a 30  40 aCTCCAACGAt 30 |
| --- | --- |

|  | 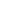 | | | FINGER | HELIX | TRIPLET | REFERENCE NUMBER | SOURCE | | --- | --- | --- | --- | --- | | F1 | TKQILGR | [GCT](http://bindr.gdcb.iastate.edu:8080/ZiFDB/controller/searchFinger?target=GCT) | - | CoDA | | F2 | HKSSLTR | [GTT](http://bindr.gdcb.iastate.edu:8080/ZiFDB/controller/searchFinger?target=GTT) | - | CoDA | | F3 | RHDQLTR | [GAG](http://bindr.gdcb.iastate.edu:8080/ZiFDB/controller/searchFinger?target=GAG) | - | CoDA |   [ZF DNA Sequence](javascript:CoDAPopupArrayWindow("ZFA-unknown-1","TKQILGR","HKSSLTR","RHDQLTR")) | |
| --- | --- | --- | --- | --- | --- | --- | --- | --- | --- | --- | --- | --- | --- | --- | --- | --- | --- | --- | --- | --- | --- | --- | --- | --- |
| [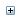](javascript:TreeView_ToggleNode(ctl00_ContentPlaceHolder1_tree12_Data,2,document.getElementById('ctl00_ContentPlaceHolder1_tree12n2'),'%20',document.getElementById('ctl00_ContentPlaceHolder1_tree12n2Nodes'))) | | ZFA-unknown-2 51 t[GGTGTCTGT](http://bindr.gdcb.iastate.edu:8080/ZiFDB/controller/searchArray?site=TGTGTCGGT)t 41  51 aCCACAGACAa 41 | |  |

|  | 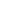 | | | FINGER | HELIX | TRIPLET | REFERENCE NUMBER | SOURCE | | --- | --- | --- | --- | --- | | F1 | RKQHLVL | [TGT](http://bindr.gdcb.iastate.edu:8080/ZiFDB/controller/searchFinger?target=TGT) | - | CoDA | | F2 | DHSSLKR | [GTC](http://bindr.gdcb.iastate.edu:8080/ZiFDB/controller/searchFinger?target=GTC) | - | CoDA | | F3 | QPHHLPR | [GGT](http://bindr.gdcb.iastate.edu:8080/ZiFDB/controller/searchFinger?target=GGT) | - | CoDA |   [ZF DNA Sequence](javascript:CoDAPopupArrayWindow("ZFA-unknown-2","RKQHLVL","DHSSLKR","QPHHLPR")) | |
| --- | --- | --- | --- | --- | --- | --- | --- | --- | --- | --- | --- | --- | --- | --- | --- | --- | --- | --- | --- | --- | --- | --- | --- | --- |
| [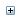](javascript:TreeView_ToggleNode(ctl00_ContentPlaceHolder1_tree12_Data,4,document.getElementById('ctl00_ContentPlaceHolder1_tree12n4'),'%20',document.getElementById('ctl00_ContentPlaceHolder1_tree12n4Nodes'))) | | **ZFA-unknown-3 70 a**[**GGAGAAGTC**](http://bindr.gdcb.iastate.edu:8080/ZiFDB/controller/searchArray?site=GTCGAAGGA)**t 80  70 tCCTCTTCAGa 80** | |  |

|  | 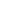 | | | FINGER | HELIX | TRIPLET | REFERENCE NUMBER | SOURCE | | --- | --- | --- | --- | --- | | F1 | TSTLLNR | [GTC](http://bindr.gdcb.iastate.edu:8080/ZiFDB/controller/searchFinger?target=GTC) | - | CoDA | | F2 | QQTNLTR | [GAA](http://bindr.gdcb.iastate.edu:8080/ZiFDB/controller/searchFinger?target=GAA) | - | CoDA | | F3 | QTTHLSR | [GGA](http://bindr.gdcb.iastate.edu:8080/ZiFDB/controller/searchFinger?target=GGA) | - | CoDA |   [ZF DNA Sequence](javascript:CoDAPopupArrayWindow("ZFA-unknown-3","TSTLLNR","QQTNLTR","QTTHLSR")) | |
| --- | --- | --- | --- | --- | --- | --- | --- | --- | --- | --- | --- | --- | --- | --- | --- | --- | --- | --- | --- | --- | --- | --- | --- | --- |
| [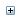](javascript:TreeView_ToggleNode(ctl00_ContentPlaceHolder1_tree12_Data,6,document.getElementById('ctl00_ContentPlaceHolder1_tree12n6'),'%20',document.getElementById('ctl00_ContentPlaceHolder1_tree12n6Nodes'))) | | ZFA-unknown-4 73 a[GAAGTC**TGC**](http://bindr.gdcb.iastate.edu:8080/ZiFDB/controller/searchArray?site=TGCGTCGAA)c 83  73 tCTTCAGACGg 83 | |  |

|  | 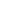 | | | FINGER | HELIX | TRIPLET | REFERENCE NUMBER | SOURCE | | --- | --- | --- | --- | --- | | F1 | RKRNLIM | [**TGC**](http://bindr.gdcb.iastate.edu:8080/ZiFDB/controller/searchFinger?target=TGC) | - | CoDA | | F2 | DHSSLKR | [GTC](http://bindr.gdcb.iastate.edu:8080/ZiFDB/controller/searchFinger?target=GTC) | - | CoDA | | F3 | QHPNLTR | [GAA](http://bindr.gdcb.iastate.edu:8080/ZiFDB/controller/searchFinger?target=GAA) | - | CoDA |   [ZF DNA Sequence](javascript:CoDAPopupArrayWindow("ZFA-unknown-4","RKRNLIM","DHSSLKR","QHPNLTR")) | |
| --- | --- | --- | --- | --- | --- | --- | --- | --- | --- | --- | --- | --- | --- | --- | --- | --- | --- | --- | --- | --- | --- | --- | --- | --- |
| [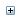](javascript:TreeView_ToggleNode(ctl00_ContentPlaceHolder1_tree12_Data,8,document.getElementById('ctl00_ContentPlaceHolder1_tree12n8'),'%20',document.getElementById('ctl00_ContentPlaceHolder1_tree12n8Nodes'))) | | ZFA-unknown-5 92 g[GCAGTAACG](http://bindr.gdcb.iastate.edu:8080/ZiFDB/controller/searchArray?site=ACGGTAGCA)g 82  92 cCGTCATTGCc 82 | |  |

|  | 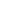 | | | FINGER | HELIX | TRIPLET | REFERENCE NUMBER | SOURCE | | --- | --- | --- | --- | --- | | F1 | RNITLVR | [ACG](http://bindr.gdcb.iastate.edu:8080/ZiFDB/controller/searchFinger?target=ACG) | - | CoDA | | F2 | QRSSLVR | [GTA](http://bindr.gdcb.iastate.edu:8080/ZiFDB/controller/searchFinger?target=GTA) | - | CoDA | | F3 | QDNTLRR | [GCA](http://bindr.gdcb.iastate.edu:8080/ZiFDB/controller/searchFinger?target=GCA) | - | CoDA |   [ZF DNA Sequence](javascript:CoDAPopupArrayWindow("ZFA-unknown-5","RNITLVR","QRSSLVR","QDNTLRR")) | |
| --- | --- | --- | --- | --- | --- | --- | --- | --- | --- | --- | --- | --- | --- | --- | --- | --- | --- | --- | --- | --- | --- | --- | --- | --- |
| [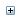](javascript:TreeView_ToggleNode(ctl00_ContentPlaceHolder1_tree12_Data,10,document.getElementById('ctl00_ContentPlaceHolder1_tree12n10'),'%20',document.getElementById('ctl00_ContentPlaceHolder1_tree12n10Nodes'))) | | ZFA-unknown-6 93 c[TGTGGGGCA](http://bindr.gdcb.iastate.edu:8080/ZiFDB/controller/searchArray?site=GCAGGGTGT)a 103  93 gACACCCCGTt 103 | |  |

|  | 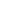 | | | FINGER | HELIX | TRIPLET | REFERENCE NUMBER | SOURCE | | --- | --- | --- | --- | --- | | F1 | DRSQLAR | [GCA](http://bindr.gdcb.iastate.edu:8080/ZiFDB/controller/searchFinger?target=GCA) | - | CoDA | | F2 | RREHLVR | [GGG](http://bindr.gdcb.iastate.edu:8080/ZiFDB/controller/searchFinger?target=GGG) | - | CoDA | | F3 | QRHGLSS | [TGT](http://bindr.gdcb.iastate.edu:8080/ZiFDB/controller/searchFinger?target=TGT) | - | CoDA |   [ZF DNA Sequence](javascript:CoDAPopupArrayWindow("ZFA-unknown-6","DRSQLAR","RREHLVR","QRHGLSS")) | |
| --- | --- | --- | --- | --- | --- | --- | --- | --- | --- | --- | --- | --- | --- | --- | --- | --- | --- | --- | --- | --- | --- | --- | --- | --- |
| [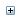](javascript:TreeView_ToggleNode(ctl00_ContentPlaceHolder1_tree12_Data,12,document.getElementById('ctl00_ContentPlaceHolder1_tree12n12'),'%20',document.getElementById('ctl00_ContentPlaceHolder1_tree12n12Nodes'))) | | ZFA-unknown-7 96 t[GGGGCAAGG](http://bindr.gdcb.iastate.edu:8080/ZiFDB/controller/searchArray?site=AGGGCAGGG)t 106  96 aCCCCGTTCCa 106 | |  |

|  | 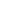 | | | FINGER | HELIX | TRIPLET | REFERENCE NUMBER | SOURCE | | --- | --- | --- | --- | --- | | F1 | RNEHLKV | [AGG](http://bindr.gdcb.iastate.edu:8080/ZiFDB/controller/searchFinger?target=AGG) | - | CoDA | | F2 | QSTTLKR | [GCA](http://bindr.gdcb.iastate.edu:8080/ZiFDB/controller/searchFinger?target=GCA) | - | CoDA | | F3 | RTEHLAR | [GGG](http://bindr.gdcb.iastate.edu:8080/ZiFDB/controller/searchFinger?target=GGG) | - | CoDA |   [ZF DNA Sequence](javascript:CoDAPopupArrayWindow("ZFA-unknown-7","RNEHLKV","QSTTLKR","RTEHLAR")) | |
| --- | --- | --- | --- | --- | --- | --- | --- | --- | --- | --- | --- | --- | --- | --- | --- | --- | --- | --- | --- | --- | --- | --- | --- | --- |
| [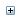](javascript:TreeView_ToggleNode(ctl00_ContentPlaceHolder1_tree12_Data,14,document.getElementById('ctl00_ContentPlaceHolder1_tree12n14'),'%20',document.getElementById('ctl00_ContentPlaceHolder1_tree12n14Nodes'))) | | ZFA-unknown-8 110 c[GTGGATGAA](http://bindr.gdcb.iastate.edu:8080/ZiFDB/controller/searchArray?site=GAAGATGTG)g 120  110 gCACCTACTTc 120 | |  |

|  | 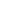 | | | FINGER | HELIX | TRIPLET | REFERENCE NUMBER | SOURCE | | --- | --- | --- | --- | --- | | F1 | RKPNLLR | [GAA](http://bindr.gdcb.iastate.edu:8080/ZiFDB/controller/searchFinger?target=GAA) | - | CoDA | | F2 | VRHNLTR | [GAT](http://bindr.gdcb.iastate.edu:8080/ZiFDB/controller/searchFinger?target=GAT) | - | CoDA | | F3 | RRAALGP | [GTG](http://bindr.gdcb.iastate.edu:8080/ZiFDB/controller/searchFinger?target=GTG) | - | CoDA |   [ZF DNA Sequence](javascript:CoDAPopupArrayWindow("ZFA-unknown-8","RKPNLLR","VRHNLTR","RRAALGP")) | |
| --- | --- | --- | --- | --- | --- | --- | --- | --- | --- | --- | --- | --- | --- | --- | --- | --- | --- | --- | --- | --- | --- | --- | --- | --- |
| [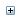](javascript:TreeView_ToggleNode(ctl00_ContentPlaceHolder1_tree12_Data,16,document.getElementById('ctl00_ContentPlaceHolder1_tree12n16'),'%20',document.getElementById('ctl00_ContentPlaceHolder1_tree12n16Nodes'))) | | ZFA-unknown-9 113 g[GATGAAGTT](http://bindr.gdcb.iastate.edu:8080/ZiFDB/controller/searchArray?site=GTTGAAGAT)g 123  113 cCTACTTCAAc 123 | |  |

|  | 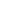 | | | FINGER | HELIX | TRIPLET | REFERENCE NUMBER | SOURCE | | --- | --- | --- | --- | --- | | F1 | TSTLLKR | [GTT](http://bindr.gdcb.iastate.edu:8080/ZiFDB/controller/searchFinger?target=GTT) | - | CoDA | | F2 | QQTNLTR | [GAA](http://bindr.gdcb.iastate.edu:8080/ZiFDB/controller/searchFinger?target=GAA) | - | CoDA | | F3 | VGSNLTR | [GAT](http://bindr.gdcb.iastate.edu:8080/ZiFDB/controller/searchFinger?target=GAT) | - | CoDA |   [ZF DNA Sequence](javascript:CoDAPopupArrayWindow("ZFA-unknown-9","TSTLLKR","QQTNLTR","VGSNLTR")) | |
| --- | --- | --- | --- | --- | --- | --- | --- | --- | --- | --- | --- | --- | --- | --- | --- | --- | --- | --- | --- | --- | --- | --- | --- | --- |
| [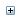](javascript:TreeView_ToggleNode(ctl00_ContentPlaceHolder1_tree12_Data,18,document.getElementById('ctl00_ContentPlaceHolder1_tree12n18'),'%20',document.getElementById('ctl00_ContentPlaceHolder1_tree12n18Nodes'))) | | ZFA-unknown-10 116 t[GAAGTTGGT](http://bindr.gdcb.iastate.edu:8080/ZiFDB/controller/searchArray?site=GGTGTTGAA)g 126  116 aCTTCAACCAc 126 | |  |

|  | 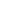 | | | FINGER | HELIX | TRIPLET | REFERENCE NUMBER | SOURCE | | --- | --- | --- | --- | --- | | F1 | RRQKLTI | [GGT](http://bindr.gdcb.iastate.edu:8080/ZiFDB/controller/searchFinger?target=GGT) | - | CoDA | | F2 | HKSSLTR | [GTT](http://bindr.gdcb.iastate.edu:8080/ZiFDB/controller/searchFinger?target=GTT) | - | CoDA | | F3 | QTNNLGR | [GAA](http://bindr.gdcb.iastate.edu:8080/ZiFDB/controller/searchFinger?target=GAA) | - | CoDA |   [ZF DNA Sequence](javascript:CoDAPopupArrayWindow("ZFA-unknown-10","RRQKLTI","HKSSLTR","QTNNLGR")) | |
| --- | --- | --- | --- | --- | --- | --- | --- | --- | --- | --- | --- | --- | --- | --- | --- | --- | --- | --- | --- | --- | --- | --- | --- | --- |
| [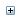](javascript:TreeView_ToggleNode(ctl00_ContentPlaceHolder1_tree12_Data,20,document.getElementById('ctl00_ContentPlaceHolder1_tree12n20'),'%20',document.getElementById('ctl00_ContentPlaceHolder1_tree12n20Nodes'))) | | ZFA-unknown-11 119 a[GTTGGTGGT](http://bindr.gdcb.iastate.edu:8080/ZiFDB/controller/searchArray?site=GGTGGTGTT)g 129  119 tCAACCACCAc 129 | |  |

|  | 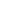 | | | FINGER | HELIX | TRIPLET | REFERENCE NUMBER | SOURCE | | --- | --- | --- | --- | --- | | F1 | MKHHLAR | [GGT](http://bindr.gdcb.iastate.edu:8080/ZiFDB/controller/searchFinger?target=GGT) | - | CoDA | | F2 | EAHHLSR | [GGT](http://bindr.gdcb.iastate.edu:8080/ZiFDB/controller/searchFinger?target=GGT) | - | CoDA | | F3 | IRTSLKR | [GTT](http://bindr.gdcb.iastate.edu:8080/ZiFDB/controller/searchFinger?target=GTT) | - | CoDA |   [ZF DNA Sequence](javascript:CoDAPopupArrayWindow("ZFA-unknown-11","MKHHLAR","EAHHLSR","IRTSLKR")) | |
| --- | --- | --- | --- | --- | --- | --- | --- | --- | --- | --- | --- | --- | --- | --- | --- | --- | --- | --- | --- | --- | --- | --- | --- | --- |
| [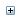](javascript:TreeView_ToggleNode(ctl00_ContentPlaceHolder1_tree12_Data,22,document.getElementById('ctl00_ContentPlaceHolder1_tree12n22'),'%20',document.getElementById('ctl00_ContentPlaceHolder1_tree12n22Nodes'))) | | ZFA-unknown-12 122 t[GGTGGTGAG](http://bindr.gdcb.iastate.edu:8080/ZiFDB/controller/searchArray?site=GAGGGTGGT)g 132  122 aCCACCACTCc 132 | |  |

|  | 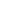 | | | FINGER | HELIX | TRIPLET | REFERENCE NUMBER | SOURCE | | --- | --- | --- | --- | --- | | F1 | RNTNLTR | [GAG](http://bindr.gdcb.iastate.edu:8080/ZiFDB/controller/searchFinger?target=GAG) | - | CoDA | | F2 | EAHHLSR | [GGT](http://bindr.gdcb.iastate.edu:8080/ZiFDB/controller/searchFinger?target=GGT) | - | CoDA | | F3 | IRHHLKR | [GGT](http://bindr.gdcb.iastate.edu:8080/ZiFDB/controller/searchFinger?target=GGT) | - | CoDA |   [ZF DNA Sequence](javascript:CoDAPopupArrayWindow("ZFA-unknown-12","RNTNLTR","EAHHLSR","IRHHLKR")) | |
| --- | --- | --- | --- | --- | --- | --- | --- | --- | --- | --- | --- | --- | --- | --- | --- | --- | --- | --- | --- | --- | --- | --- | --- | --- |
| [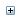](javascript:TreeView_ToggleNode(ctl00_ContentPlaceHolder1_tree12_Data,24,document.getElementById('ctl00_ContentPlaceHolder1_tree12n24'),'%20',document.getElementById('ctl00_ContentPlaceHolder1_tree12n24Nodes'))) | | ZFA-unknown-13 125 t[GGTGAGGCC](http://bindr.gdcb.iastate.edu:8080/ZiFDB/controller/searchArray?site=GCCGAGGGT)c 135  125 aCCACTCCGGg 135 | |  |

|  | 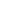 | | | FINGER | HELIX | TRIPLET | REFERENCE NUMBER | SOURCE | | --- | --- | --- | --- | --- | | F1 | VRKDLTR | [GCC](http://bindr.gdcb.iastate.edu:8080/ZiFDB/controller/searchFinger?target=GCC) | - | CoDA | | F2 | RQDNLGR | [GAG](http://bindr.gdcb.iastate.edu:8080/ZiFDB/controller/searchFinger?target=GAG) | - | CoDA | | F3 | VKHGLGR | [GGT](http://bindr.gdcb.iastate.edu:8080/ZiFDB/controller/searchFinger?target=GGT) | - | CoDA |   [ZF DNA Sequence](javascript:CoDAPopupArrayWindow("ZFA-unknown-13","VRKDLTR","RQDNLGR","VKHGLGR")) | |
| --- | --- | --- | --- | --- | --- | --- | --- | --- | --- | --- | --- | --- | --- | --- | --- | --- | --- | --- | --- | --- | --- | --- | --- | --- |
| [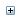](javascript:TreeView_ToggleNode(ctl00_ContentPlaceHolder1_tree12_Data,26,document.getElementById('ctl00_ContentPlaceHolder1_tree12n26'),'%20',document.getElementById('ctl00_ContentPlaceHolder1_tree12n26Nodes'))) | | ZFA-unknown-14 135 c[TGGGCAGGT](http://bindr.gdcb.iastate.edu:8080/ZiFDB/controller/searchArray?site=GGTGCATGG)t 145  135 gACCCGTCCAa 145 | |  |

|  | 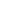 | | | FINGER | HELIX | TRIPLET | REFERENCE NUMBER | SOURCE | | --- | --- | --- | --- | --- | | F1 | TTTKLAI | [GGT](http://bindr.gdcb.iastate.edu:8080/ZiFDB/controller/searchFinger?target=GGT) | - | CoDA | | F2 | QSTTLKR | [GCA](http://bindr.gdcb.iastate.edu:8080/ZiFDB/controller/searchFinger?target=GCA) | - | CoDA | | F3 | RSDHLSL | [TGG](http://bindr.gdcb.iastate.edu:8080/ZiFDB/controller/searchFinger?target=TGG) | - | CoDA |   [ZF DNA Sequence](javascript:CoDAPopupArrayWindow("ZFA-unknown-14","TTTKLAI","QSTTLKR","RSDHLSL")) | |
| --- | --- | --- | --- | --- | --- | --- | --- | --- | --- | --- | --- | --- | --- | --- | --- | --- | --- | --- | --- | --- | --- | --- | --- | --- |
| [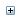](javascript:TreeView_ToggleNode(ctl00_ContentPlaceHolder1_tree12_Data,28,document.getElementById('ctl00_ContentPlaceHolder1_tree12n28'),'%20',document.getElementById('ctl00_ContentPlaceHolder1_tree12n28Nodes'))) | | ZFA-unknown-15 169 t[TAAGGAGAC](http://bindr.gdcb.iastate.edu:8080/ZiFDB/controller/searchArray?site=GACGGATAA)c 179  169 aATTCCTCTGg 179 | |  |

|  | 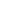 | | | FINGER | HELIX | TRIPLET | REFERENCE NUMBER | SOURCE | | --- | --- | --- | --- | --- | | F1 | DEANLRR | [GAC](http://bindr.gdcb.iastate.edu:8080/ZiFDB/controller/searchFinger?target=GAC) | - | CoDA | | F2 | QSAHLKR | [GGA](http://bindr.gdcb.iastate.edu:8080/ZiFDB/controller/searchFinger?target=GGA) | - | CoDA | | F3 | QRGNLNM | [TAA](http://bindr.gdcb.iastate.edu:8080/ZiFDB/controller/searchFinger?target=TAA) | - | CoDA |   [ZF DNA Sequence](javascript:CoDAPopupArrayWindow("ZFA-unknown-15","DEANLRR","QSAHLKR","QRGNLNM")) | |
| --- | --- | --- | --- | --- | --- | --- | --- | --- | --- | --- | --- | --- | --- | --- | --- | --- | --- | --- | --- | --- | --- | --- | --- | --- |
| [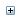](javascript:TreeView_ToggleNode(ctl00_ContentPlaceHolder1_tree12_Data,30,document.getElementById('ctl00_ContentPlaceHolder1_tree12n30'),'%20',document.getElementById('ctl00_ContentPlaceHolder1_tree12n30Nodes'))) | | ZFA-unknown-16 188 c[TGGGCATGT](http://bindr.gdcb.iastate.edu:8080/ZiFDB/controller/searchArray?site=TGTGCATGG)g 198  188 gACCCGTACAc 198 | |  |

|  | 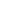 | | | FINGER | HELIX | TRIPLET | REFERENCE NUMBER | SOURCE | | --- | --- | --- | --- | --- | | F1 | RRQHLQY | [TGT](http://bindr.gdcb.iastate.edu:8080/ZiFDB/controller/searchFinger?target=TGT) | - | CoDA | | F2 | QSTTLKR | [GCA](http://bindr.gdcb.iastate.edu:8080/ZiFDB/controller/searchFinger?target=GCA) | - | CoDA | | F3 | RSDHLSL | [TGG](http://bindr.gdcb.iastate.edu:8080/ZiFDB/controller/searchFinger?target=TGG) | - | CoDA |   [ZF DNA Sequence](javascript:CoDAPopupArrayWindow("ZFA-unknown-16","RRQHLQY","QSTTLKR","RSDHLSL")) | |
| --- | --- | --- | --- | --- | --- | --- | --- | --- | --- | --- | --- | --- | --- | --- | --- | --- | --- | --- | --- | --- | --- | --- | --- | --- |
| [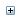](javascript:TreeView_ToggleNode(ctl00_ContentPlaceHolder1_tree12_Data,32,document.getElementById('ctl00_ContentPlaceHolder1_tree12n32'),'%20',document.getElementById('ctl00_ContentPlaceHolder1_tree12n32Nodes'))) | | ZFA-unknown-17 194 a[TGTGGAGAC](http://bindr.gdcb.iastate.edu:8080/ZiFDB/controller/searchArray?site=GACGGATGT)a 204  194 tACACCTCTGt 204 | |  |

|  | 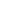 | | | FINGER | HELIX | TRIPLET | REFERENCE NUMBER | SOURCE | | --- | --- | --- | --- | --- | | F1 | DEANLRR | [GAC](http://bindr.gdcb.iastate.edu:8080/ZiFDB/controller/searchFinger?target=GAC) | - | CoDA | | F2 | QSAHLKR | [GGA](http://bindr.gdcb.iastate.edu:8080/ZiFDB/controller/searchFinger?target=GGA) | - | CoDA | | F3 | QPHGLAH | [TGT](http://bindr.gdcb.iastate.edu:8080/ZiFDB/controller/searchFinger?target=TGT) | - | CoDA |   [ZF DNA Sequence](javascript:CoDAPopupArrayWindow("ZFA-unknown-17","DEANLRR","QSAHLKR","QPHGLAH")) | |
| --- | --- | --- | --- | --- | --- | --- | --- | --- | --- | --- | --- | --- | --- | --- | --- | --- | --- | --- | --- | --- | --- | --- | --- | --- |
| [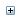](javascript:TreeView_ToggleNode(ctl00_ContentPlaceHolder1_tree12_Data,34,document.getElementById('ctl00_ContentPlaceHolder1_tree12n34'),'%20',document.getElementById('ctl00_ContentPlaceHolder1_tree12n34Nodes'))) | | ZFA-unknown-18 250 a[TAGGCAGAG](http://bindr.gdcb.iastate.edu:8080/ZiFDB/controller/searchArray?site=GAGGCATAG)a 240  250 tATCCGTCTCt 240 | |  |

|  | 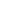 | | | FINGER | HELIX | TRIPLET | REFERENCE NUMBER | SOURCE | | --- | --- | --- | --- | --- | | F1 | KHSNLAR | [GAG](http://bindr.gdcb.iastate.edu:8080/ZiFDB/controller/searchFinger?target=GAG) | - | CoDA | | F2 | QSTTLKR | [GCA](http://bindr.gdcb.iastate.edu:8080/ZiFDB/controller/searchFinger?target=GCA) | - | CoDA | | F3 | RRDGLAG | [TAG](http://bindr.gdcb.iastate.edu:8080/ZiFDB/controller/searchFinger?target=TAG) | - | CoDA |   [ZF DNA Sequence](javascript:CoDAPopupArrayWindow("ZFA-unknown-18","KHSNLAR","QSTTLKR","RRDGLAG")) | |
| --- | --- | --- | --- | --- | --- | --- | --- | --- | --- | --- | --- | --- | --- | --- | --- | --- | --- | --- | --- | --- | --- | --- | --- | --- |
| [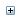](javascript:TreeView_ToggleNode(ctl00_ContentPlaceHolder1_tree12_Data,36,document.getElementById('ctl00_ContentPlaceHolder1_tree12n36'),'%20',document.getElementById('ctl00_ContentPlaceHolder1_tree12n36Nodes'))) | | ZFA-unknown-19 269 t[TAGGCTGCT](http://bindr.gdcb.iastate.edu:8080/ZiFDB/controller/searchArray?site=GCTGCTTAG)g 279  269 aATCCGACGAc 279 | |  |

|  | 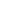 | | | FINGER | HELIX | TRIPLET | REFERENCE NUMBER | SOURCE | | --- | --- | --- | --- | --- | | F1 | MKNTLTR | [GCT](http://bindr.gdcb.iastate.edu:8080/ZiFDB/controller/searchFinger?target=GCT) | - | CoDA | | F2 | QRSDLTR | [GCT](http://bindr.gdcb.iastate.edu:8080/ZiFDB/controller/searchFinger?target=GCT) | - | CoDA | | F3 | RRDNLPK | [TAG](http://bindr.gdcb.iastate.edu:8080/ZiFDB/controller/searchFinger?target=TAG) | - | CoDA |   [ZF DNA Sequence](javascript:CoDAPopupArrayWindow("ZFA-unknown-19","MKNTLTR","QRSDLTR","RRDNLPK")) | |
| --- | --- | --- | --- | --- | --- | --- | --- | --- | --- | --- | --- | --- | --- | --- | --- | --- | --- | --- | --- | --- | --- | --- | --- | --- |
| [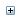](javascript:TreeView_ToggleNode(ctl00_ContentPlaceHolder1_tree12_Data,38,document.getElementById('ctl00_ContentPlaceHolder1_tree12n38'),'%20',document.getElementById('ctl00_ContentPlaceHolder1_tree12n38Nodes'))) | | ZFA-unknown-20 272 g[GCTGCTGGT](http://bindr.gdcb.iastate.edu:8080/ZiFDB/controller/searchArray?site=GGTGCTGCT)g 282  272 cCGACGACCAc 282 | |  |

|  | 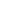 | | | FINGER | HELIX | TRIPLET | REFERENCE NUMBER | SOURCE | | --- | --- | --- | --- | --- | | F1 | RRQKLTI | [GGT](http://bindr.gdcb.iastate.edu:8080/ZiFDB/controller/searchFinger?target=GGT) | - | CoDA | | F2 | QRSDLTR | [GCT](http://bindr.gdcb.iastate.edu:8080/ZiFDB/controller/searchFinger?target=GCT) | - | CoDA | | F3 | LRASLRR | [GCT](http://bindr.gdcb.iastate.edu:8080/ZiFDB/controller/searchFinger?target=GCT) | - | CoDA |   [ZF DNA Sequence](javascript:CoDAPopupArrayWindow("ZFA-unknown-20","RRQKLTI","QRSDLTR","LRASLRR")) | |
| --- | --- | --- | --- | --- | --- | --- | --- | --- | --- | --- | --- | --- | --- | --- | --- | --- | --- | --- | --- | --- | --- | --- | --- | --- |
| [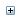](javascript:TreeView_ToggleNode(ctl00_ContentPlaceHolder1_tree12_Data,40,document.getElementById('ctl00_ContentPlaceHolder1_tree12n40'),'%20',document.getElementById('ctl00_ContentPlaceHolder1_tree12n40Nodes'))) | | ZFA-unknown-21 275 t[GCTGGTGGT](http://bindr.gdcb.iastate.edu:8080/ZiFDB/controller/searchArray?site=GGTGGTGCT)c 285  275 aCGACCACCAg 285 | |  |

|  | 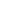 | | | FINGER | HELIX | TRIPLET | REFERENCE NUMBER | SOURCE | | --- | --- | --- | --- | --- | | F1 | MKHHLAR | [GGT](http://bindr.gdcb.iastate.edu:8080/ZiFDB/controller/searchFinger?target=GGT) | - | CoDA | | F2 | EAHHLSR | [GGT](http://bindr.gdcb.iastate.edu:8080/ZiFDB/controller/searchFinger?target=GGT) | - | CoDA | | F3 | EGSGLKR | [GCT](http://bindr.gdcb.iastate.edu:8080/ZiFDB/controller/searchFinger?target=GCT) | - | CoDA |   [ZF DNA Sequence](javascript:CoDAPopupArrayWindow("ZFA-unknown-21","MKHHLAR","EAHHLSR","EGSGLKR")) | |
| --- | --- | --- | --- | --- | --- | --- | --- | --- | --- | --- | --- | --- | --- | --- | --- | --- | --- | --- | --- | --- | --- | --- | --- | --- |
| [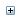](javascript:TreeView_ToggleNode(ctl00_ContentPlaceHolder1_tree12_Data,42,document.getElementById('ctl00_ContentPlaceHolder1_tree12n42'),'%20',document.getElementById('ctl00_ContentPlaceHolder1_tree12n42Nodes'))) | | ZFA-unknown-22 336 a[GGAGTGGAC](http://bindr.gdcb.iastate.edu:8080/ZiFDB/controller/searchArray?site=GACGTGGGA)a 326  336 tCCTCACCTGt 326 | |  |

|  | 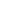 | | | FINGER | HELIX | TRIPLET | REFERENCE NUMBER | SOURCE | | --- | --- | --- | --- | --- | | F1 | DEANLRR | [GAC](http://bindr.gdcb.iastate.edu:8080/ZiFDB/controller/searchFinger?target=GAC) | - | CoDA | | F2 | RREVLEN | [GTG](http://bindr.gdcb.iastate.edu:8080/ZiFDB/controller/searchFinger?target=GTG) | - | CoDA | | F3 | QKPHLSR | [GGA](http://bindr.gdcb.iastate.edu:8080/ZiFDB/controller/searchFinger?target=GGA) | - | CoDA |   [ZF DNA Sequence](javascript:CoDAPopupArrayWindow("ZFA-unknown-22","DEANLRR","RREVLEN","QKPHLSR")) | |
| --- | --- | --- | --- | --- | --- | --- | --- | --- | --- | --- | --- | --- | --- | --- | --- | --- | --- | --- | --- | --- | --- | --- | --- | --- |
| [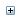](javascript:TreeView_ToggleNode(ctl00_ContentPlaceHolder1_tree12_Data,44,document.getElementById('ctl00_ContentPlaceHolder1_tree12n44'),'%20',document.getElementById('ctl00_ContentPlaceHolder1_tree12n44Nodes'))) | | ZFA-unknown-23 336 t[GATGCTGTT](http://bindr.gdcb.iastate.edu:8080/ZiFDB/controller/searchArray?site=GTTGCTGAT)a 346  336 aCTACGACAAt 346 | |  |

|  | 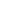 | | | FINGER | HELIX | TRIPLET | REFERENCE NUMBER | SOURCE | | --- | --- | --- | --- | --- | | F1 | QATLLRR | [GTT](http://bindr.gdcb.iastate.edu:8080/ZiFDB/controller/searchFinger?target=GTT) | - | CoDA | | F2 | QRSDLTR | [GCT](http://bindr.gdcb.iastate.edu:8080/ZiFDB/controller/searchFinger?target=GCT) | - | CoDA | | F3 | LTHNLRR | [GAT](http://bindr.gdcb.iastate.edu:8080/ZiFDB/controller/searchFinger?target=GAT) | - | CoDA |   [ZF DNA Sequence](javascript:CoDAPopupArrayWindow("ZFA-unknown-23","QATLLRR","QRSDLTR","LTHNLRR")) | |
| --- | --- | --- | --- | --- | --- | --- | --- | --- | --- | --- | --- | --- | --- | --- | --- | --- | --- | --- | --- | --- | --- | --- | --- | --- |
| [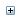](javascript:TreeView_ToggleNode(ctl00_ContentPlaceHolder1_tree12_Data,46,document.getElementById('ctl00_ContentPlaceHolder1_tree12n46'),'%20',document.getElementById('ctl00_ContentPlaceHolder1_tree12n46Nodes'))) | | ZFA-unknown-24 359 a[GGTGAAGGC](http://bindr.gdcb.iastate.edu:8080/ZiFDB/controller/searchArray?site=GGCGAAGGT)t 369  359 tCCACTTCCGa 369 | |  |

|  | 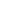 | | | FINGER | HELIX | TRIPLET | REFERENCE NUMBER | SOURCE | | --- | --- | --- | --- | --- | | F1 | TRAKLHI | [GGC](http://bindr.gdcb.iastate.edu:8080/ZiFDB/controller/searchFinger?target=GGC) | - | CoDA | | F2 | QQTNLTR | [GAA](http://bindr.gdcb.iastate.edu:8080/ZiFDB/controller/searchFinger?target=GAA) | - | CoDA | | F3 | IRHHLKR | [GGT](http://bindr.gdcb.iastate.edu:8080/ZiFDB/controller/searchFinger?target=GGT) | - | CoDA |   [ZF DNA Sequence](javascript:CoDAPopupArrayWindow("ZFA-unknown-24","TRAKLHI","QQTNLTR","IRHHLKR")) | |
| --- | --- | --- | --- | --- | --- | --- | --- | --- | --- | --- | --- | --- | --- | --- | --- | --- | --- | --- | --- | --- | --- | --- | --- | --- |
| [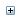](javascript:TreeView_ToggleNode(ctl00_ContentPlaceHolder1_tree12_Data,48,document.getElementById('ctl00_ContentPlaceHolder1_tree12n48'),'%20',document.getElementById('ctl00_ContentPlaceHolder1_tree12n48Nodes'))) | | ZFA-unknown-25 427 t[GAGGTTGTC](http://bindr.gdcb.iastate.edu:8080/ZiFDB/controller/searchArray?site=GTCGTTGAG)c 417  427 aCTCCAACAGg 417 | |  |

|  | 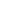 | | | FINGER | HELIX | TRIPLET | REFERENCE NUMBER | SOURCE | | --- | --- | --- | --- | --- | | F1 | TKKILTV | [GTC](http://bindr.gdcb.iastate.edu:8080/ZiFDB/controller/searchFinger?target=GTC) | - | CoDA | | F2 | HKSSLTR | [GTT](http://bindr.gdcb.iastate.edu:8080/ZiFDB/controller/searchFinger?target=GTT) | - | CoDA | | F3 | RHDQLTR | [GAG](http://bindr.gdcb.iastate.edu:8080/ZiFDB/controller/searchFinger?target=GAG) | - | CoDA |   [ZF DNA Sequence](javascript:CoDAPopupArrayWindow("ZFA-unknown-25","TKKILTV","HKSSLTR","RHDQLTR")) | |
| --- | --- | --- | --- | --- | --- | --- | --- | --- | --- | --- | --- | --- | --- | --- | --- | --- | --- | --- | --- | --- | --- | --- | --- | --- |
| [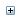](javascript:TreeView_ToggleNode(ctl00_ContentPlaceHolder1_tree12_Data,50,document.getElementById('ctl00_ContentPlaceHolder1_tree12n50'),'%20',document.getElementById('ctl00_ContentPlaceHolder1_tree12n50Nodes'))) | | ZFA-unknown-26 473 t[GCAGCTTGT](http://bindr.gdcb.iastate.edu:8080/ZiFDB/controller/searchArray?site=TGTGCTGCA)c 463  473 aCGTCGAACAg 463 | |  |

|  | 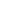 | | | FINGER | HELIX | TRIPLET | REFERENCE NUMBER | SOURCE | | --- | --- | --- | --- | --- | | F1 | KRQHLEY | [TGT](http://bindr.gdcb.iastate.edu:8080/ZiFDB/controller/searchFinger?target=TGT) | - | CoDA | | F2 | QRSDLTR | [GCT](http://bindr.gdcb.iastate.edu:8080/ZiFDB/controller/searchFinger?target=GCT) | - | CoDA | | F3 | QGGTLRR | [GCA](http://bindr.gdcb.iastate.edu:8080/ZiFDB/controller/searchFinger?target=GCA) | - | CoDA |   [ZF DNA Sequence](javascript:CoDAPopupArrayWindow("ZFA-unknown-26","KRQHLEY","QRSDLTR","QGGTLRR")) | |
| --- | --- | --- | --- | --- | --- | --- | --- | --- | --- | --- | --- | --- | --- | --- | --- | --- | --- | --- | --- | --- | --- | --- | --- | --- |
| [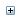](javascript:TreeView_ToggleNode(ctl00_ContentPlaceHolder1_tree12_Data,52,document.getElementById('ctl00_ContentPlaceHolder1_tree12n52'),'%20',document.getElementById('ctl00_ContentPlaceHolder1_tree12n52Nodes'))) | | ZFA-unknown-27 494 g[GGTGAGTCT](http://bindr.gdcb.iastate.edu:8080/ZiFDB/controller/searchArray?site=TCTGAGGGT)a 504  494 cCCACTCAGAt 504 | |  |

|  | 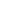 | | | FINGER | HELIX | TRIPLET | REFERENCE NUMBER | SOURCE | | --- | --- | --- | --- | --- | | F1 | SKPNLKM | [TCT](http://bindr.gdcb.iastate.edu:8080/ZiFDB/controller/searchFinger?target=TCT) | - | CoDA | | F2 | RQDNLGR | [GAG](http://bindr.gdcb.iastate.edu:8080/ZiFDB/controller/searchFinger?target=GAG) | - | CoDA | | F3 | VKHGLGR | [GGT](http://bindr.gdcb.iastate.edu:8080/ZiFDB/controller/searchFinger?target=GGT) | - | CoDA |   [ZF DNA Sequence](javascript:CoDAPopupArrayWindow("ZFA-unknown-27","SKPNLKM","RQDNLGR","VKHGLGR")) | |
| --- | --- | --- | --- | --- | --- | --- | --- | --- | --- | --- | --- | --- | --- | --- | --- | --- | --- | --- | --- | --- | --- | --- | --- | --- |
| [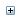](javascript:TreeView_ToggleNode(ctl00_ContentPlaceHolder1_tree12_Data,54,document.getElementById('ctl00_ContentPlaceHolder1_tree12n54'),'%20',document.getElementById('ctl00_ContentPlaceHolder1_tree12n54Nodes'))) | | ZFA-unknown-28 504 a[TGGGACGCT](http://bindr.gdcb.iastate.edu:8080/ZiFDB/controller/searchArray?site=GCTGACTGG)t 514  504 tACCCTGCGAa 514 | |  |

|  | 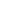 | | | FINGER | HELIX | TRIPLET | REFERENCE NUMBER | SOURCE | | --- | --- | --- | --- | --- | | F1 | VRQGLTR | [GCT](http://bindr.gdcb.iastate.edu:8080/ZiFDB/controller/searchFinger?target=GCT) | - | CoDA | | F2 | DRGNLTR | [GAC](http://bindr.gdcb.iastate.edu:8080/ZiFDB/controller/searchFinger?target=GAC) | - | CoDA | | F3 | RSDHLSL | [TGG](http://bindr.gdcb.iastate.edu:8080/ZiFDB/controller/searchFinger?target=TGG) | - | CoDA |   [ZF DNA Sequence](javascript:CoDAPopupArrayWindow("ZFA-unknown-28","VRQGLTR","DRGNLTR","RSDHLSL")) | |
| --- | --- | --- | --- | --- | --- | --- | --- | --- | --- | --- | --- | --- | --- | --- | --- | --- | --- | --- | --- | --- | --- | --- | --- | --- |
| [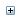](javascript:TreeView_ToggleNode(ctl00_ContentPlaceHolder1_tree12_Data,56,document.getElementById('ctl00_ContentPlaceHolder1_tree12n56'),'%20',document.getElementById('ctl00_ContentPlaceHolder1_tree12n56Nodes'))) | | ZFA-unknown-29 534 a[GAAGGGGAA](http://bindr.gdcb.iastate.edu:8080/ZiFDB/controller/searchArray?site=GAAGGGGAA)a 524  534 tCTTCCCCTTt 524 | |  |

|  | 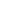 | | | FINGER | HELIX | TRIPLET | REFERENCE NUMBER | SOURCE | | --- | --- | --- | --- | --- | | F1 | QLSNLTR | [GAA](http://bindr.gdcb.iastate.edu:8080/ZiFDB/controller/searchFinger?target=GAA) | - | CoDA | | F2 | RREHLVR | [GGG](http://bindr.gdcb.iastate.edu:8080/ZiFDB/controller/searchFinger?target=GGG) | - | CoDA | | F3 | QDGNLGR | [GAA](http://bindr.gdcb.iastate.edu:8080/ZiFDB/controller/searchFinger?target=GAA) | - | CoDA |   [ZF DNA Sequence](javascript:CoDAPopupArrayWindow("ZFA-unknown-29","QLSNLTR","RREHLVR","QDGNLGR")) | |
| --- | --- | --- | --- | --- | --- | --- | --- | --- | --- | --- | --- | --- | --- | --- | --- | --- | --- | --- | --- | --- | --- | --- | --- | --- |
| [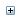](javascript:TreeView_ToggleNode(ctl00_ContentPlaceHolder1_tree12_Data,58,document.getElementById('ctl00_ContentPlaceHolder1_tree12n58'),'%20',document.getElementById('ctl00_ContentPlaceHolder1_tree12n58Nodes'))) | | ZFA-unknown-30 557 a[TAGGAAGGG](http://bindr.gdcb.iastate.edu:8080/ZiFDB/controller/searchArray?site=GGGGAATAG)g 567  557 tATCCTTCCCc 567 | |  |

|  | 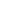 | | | FINGER | HELIX | TRIPLET | REFERENCE NUMBER | SOURCE | | --- | --- | --- | --- | --- | | F1 | KRERLDR | [GGG](http://bindr.gdcb.iastate.edu:8080/ZiFDB/controller/searchFinger?target=GGG) | - | CoDA | | F2 | QQTNLTR | [GAA](http://bindr.gdcb.iastate.edu:8080/ZiFDB/controller/searchFinger?target=GAA) | - | CoDA | | F3 | RRDHLSL | [TAG](http://bindr.gdcb.iastate.edu:8080/ZiFDB/controller/searchFinger?target=TAG) | - | CoDA |   [ZF DNA Sequence](javascript:CoDAPopupArrayWindow("ZFA-unknown-30","KRERLDR","QQTNLTR","RRDHLSL")) | |
| --- | --- | --- | --- | --- | --- | --- | --- | --- | --- | --- | --- | --- | --- | --- | --- | --- | --- | --- | --- | --- | --- | --- | --- | --- |
| [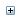](javascript:TreeView_ToggleNode(ctl00_ContentPlaceHolder1_tree12_Data,60,document.getElementById('ctl00_ContentPlaceHolder1_tree12n60'),'%20',document.getElementById('ctl00_ContentPlaceHolder1_tree12n60Nodes'))) | | ZFA-unknown-31 620 t[GTGGAAGTC](http://bindr.gdcb.iastate.edu:8080/ZiFDB/controller/searchArray?site=GTCGAAGTG)t 630  620 aCACCTTCAGa 630 | |  |

|  | 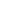 | | | FINGER | HELIX | TRIPLET | REFERENCE NUMBER | SOURCE | | --- | --- | --- | --- | --- | | F1 | TSTLLNR | [GTC](http://bindr.gdcb.iastate.edu:8080/ZiFDB/controller/searchFinger?target=GTC) | - | CoDA | | F2 | QQTNLTR | [GAA](http://bindr.gdcb.iastate.edu:8080/ZiFDB/controller/searchFinger?target=GAA) | - | CoDA | | F3 | RNVALGN | [GTG](http://bindr.gdcb.iastate.edu:8080/ZiFDB/controller/searchFinger?target=GTG) | - | CoDA |   [ZF DNA Sequence](javascript:CoDAPopupArrayWindow("ZFA-unknown-31","TSTLLNR","QQTNLTR","RNVALGN")) | |
| --- | --- | --- | --- | --- | --- | --- | --- | --- | --- | --- | --- | --- | --- | --- | --- | --- | --- | --- | --- | --- | --- | --- | --- | --- |
| [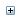](javascript:TreeView_ToggleNode(ctl00_ContentPlaceHolder1_tree12_Data,62,document.getElementById('ctl00_ContentPlaceHolder1_tree12n62'),'%20',document.getElementById('ctl00_ContentPlaceHolder1_tree12n62Nodes'))) | | ZFA-unknown-32 716 c[GGAGAAGAA](http://bindr.gdcb.iastate.edu:8080/ZiFDB/controller/searchArray?site=GAAGAAGGA)a 706  716 gCCTCTTCTTt 706 | |  |

|  | 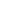 | | | FINGER | HELIX | TRIPLET | REFERENCE NUMBER | SOURCE | | --- | --- | --- | --- | --- | | F1 | QASNLTR | [GAA](http://bindr.gdcb.iastate.edu:8080/ZiFDB/controller/searchFinger?target=GAA) | - | CoDA | | F2 | QQTNLTR | [GAA](http://bindr.gdcb.iastate.edu:8080/ZiFDB/controller/searchFinger?target=GAA) | - | CoDA | | F3 | QTTHLSR | [GGA](http://bindr.gdcb.iastate.edu:8080/ZiFDB/controller/searchFinger?target=GGA) | - | CoDA |   [ZF DNA Sequence](javascript:CoDAPopupArrayWindow("ZFA-unknown-32","QASNLTR","QQTNLTR","QTTHLSR")) | |
| --- | --- | --- | --- | --- | --- | --- | --- | --- | --- | --- | --- | --- | --- | --- | --- | --- | --- | --- | --- | --- | --- | --- | --- | --- |
| [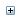](javascript:TreeView_ToggleNode(ctl00_ContentPlaceHolder1_tree12_Data,64,document.getElementById('ctl00_ContentPlaceHolder1_tree12n64'),'%20',document.getElementById('ctl00_ContentPlaceHolder1_tree12n64Nodes'))) | | ZFA-unknown-33 719 t[TGCGGAGAA](http://bindr.gdcb.iastate.edu:8080/ZiFDB/controller/searchArray?site=GAAGGATGC)g 709  719 aACGCCTCTTc 709 | |  |

|  | 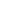 | | | FINGER | HELIX | TRIPLET | REFERENCE NUMBER | SOURCE | | --- | --- | --- | --- | --- | | F1 | QRSNLAR | [GAA](http://bindr.gdcb.iastate.edu:8080/ZiFDB/controller/searchFinger?target=GAA) | - | CoDA | | F2 | QSAHLKR | [GGA](http://bindr.gdcb.iastate.edu:8080/ZiFDB/controller/searchFinger?target=GGA) | - | CoDA | | F3 | QRRSLGH | [TGC](http://bindr.gdcb.iastate.edu:8080/ZiFDB/controller/searchFinger?target=TGC) | - | CoDA |   [ZF DNA Sequence](javascript:CoDAPopupArrayWindow("ZFA-unknown-33","QRSNLAR","QSAHLKR","QRRSLGH")) | |
| --- | --- | --- | --- | --- | --- | --- | --- | --- | --- | --- | --- | --- | --- | --- | --- | --- | --- | --- | --- | --- | --- | --- | --- | --- |
| [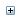](javascript:TreeView_ToggleNode(ctl00_ContentPlaceHolder1_tree12_Data,66,document.getElementById('ctl00_ContentPlaceHolder1_tree12n66'),'%20',document.getElementById('ctl00_ContentPlaceHolder1_tree12n66Nodes'))) | | ZFA-unknown-34 819 g[GCAGACTGT](http://bindr.gdcb.iastate.edu:8080/ZiFDB/controller/searchArray?site=TGTGACGCA)g 809  819 cCGTCTGACAc 809 | |  |

|  | 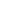 | | | FINGER | HELIX | TRIPLET | REFERENCE NUMBER | SOURCE | | --- | --- | --- | --- | --- | | F1 | RKQHLQL | [TGT](http://bindr.gdcb.iastate.edu:8080/ZiFDB/controller/searchFinger?target=TGT) | - | CoDA | | F2 | DRGNLTR | [GAC](http://bindr.gdcb.iastate.edu:8080/ZiFDB/controller/searchFinger?target=GAC) | - | CoDA | | F3 | QGNTLTR | [GCA](http://bindr.gdcb.iastate.edu:8080/ZiFDB/controller/searchFinger?target=GCA) | - | CoDA |   [ZF DNA Sequence](javascript:CoDAPopupArrayWindow("ZFA-unknown-34","RKQHLQL","DRGNLTR","QGNTLTR")) | |
| --- | --- | --- | --- | --- | --- | --- | --- | --- | --- | --- | --- | --- | --- | --- | --- | --- | --- | --- | --- | --- | --- | --- | --- | --- |
| [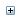](javascript:TreeView_ToggleNode(ctl00_ContentPlaceHolder1_tree12_Data,68,document.getElementById('ctl00_ContentPlaceHolder1_tree12n68'),'%20',document.getElementById('ctl00_ContentPlaceHolder1_tree12n68Nodes'))) | | ZFA-unknown-35 822 c[TAGGCAGAC](http://bindr.gdcb.iastate.edu:8080/ZiFDB/controller/searchArray?site=GACGCATAG)t 812  822 gATCCGTCTGa 812 | |  |

|  | 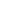 | | | FINGER | HELIX | TRIPLET | REFERENCE NUMBER | SOURCE | | --- | --- | --- | --- | --- | | F1 | EEVNLRR | [GAC](http://bindr.gdcb.iastate.edu:8080/ZiFDB/controller/searchFinger?target=GAC) | - | CoDA | | F2 | QSTTLKR | [GCA](http://bindr.gdcb.iastate.edu:8080/ZiFDB/controller/searchFinger?target=GCA) | - | CoDA | | F3 | RRDGLAG | [TAG](http://bindr.gdcb.iastate.edu:8080/ZiFDB/controller/searchFinger?target=TAG) | - | CoDA |   [ZF DNA Sequence](javascript:CoDAPopupArrayWindow("ZFA-unknown-35","EEVNLRR","QSTTLKR","RRDGLAG")) | |
| --- | --- | --- | --- | --- | --- | --- | --- | --- | --- | --- | --- | --- | --- | --- | --- | --- | --- | --- | --- | --- | --- | --- | --- | --- |
| [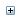](javascript:TreeView_ToggleNode(ctl00_ContentPlaceHolder1_tree12_Data,70,document.getElementById('ctl00_ContentPlaceHolder1_tree12n70'),'%20',document.getElementById('ctl00_ContentPlaceHolder1_tree12n70Nodes'))) | | ZFA-unknown-36 877 g[TAGGGAGAT](http://bindr.gdcb.iastate.edu:8080/ZiFDB/controller/searchArray?site=GATGGATAG)t 867  877 cATCCCTCTAa 867 | |  |

|  | 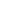 | | | FINGER | HELIX | TRIPLET | REFERENCE NUMBER | SOURCE | | --- | --- | --- | --- | --- | | F1 | TRQRLRI | [GAT](http://bindr.gdcb.iastate.edu:8080/ZiFDB/controller/searchFinger?target=GAT) | - | CoDA | | F2 | QSAHLKR | [GGA](http://bindr.gdcb.iastate.edu:8080/ZiFDB/controller/searchFinger?target=GGA) | - | CoDA | | F3 | RPEGLST | [TAG](http://bindr.gdcb.iastate.edu:8080/ZiFDB/controller/searchFinger?target=TAG) | - | CoDA |   [ZF DNA Sequence](javascript:CoDAPopupArrayWindow("ZFA-unknown-36","TRQRLRI","QSAHLKR","RPEGLST")) | |
| --- | --- | --- | --- | --- | --- | --- | --- | --- | --- | --- | --- | --- | --- | --- | --- | --- | --- | --- | --- | --- | --- | --- | --- | --- |
| [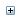](javascript:TreeView_ToggleNode(ctl00_ContentPlaceHolder1_tree12_Data,72,document.getElementById('ctl00_ContentPlaceHolder1_tree12n72'),'%20',document.getElementById('ctl00_ContentPlaceHolder1_tree12n72Nodes'))) | | ZFA-unknown-37 1201 g[TAAGAGGTT](http://bindr.gdcb.iastate.edu:8080/ZiFDB/controller/searchArray?site=GTTGAGTAA)t 1211  1201 cATTCTCCAAa 1211 | |  |

|  | 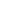 | | | FINGER | HELIX | TRIPLET | REFERENCE NUMBER | SOURCE | | --- | --- | --- | --- | --- | | F1 | TTTVLAR | [GTT](http://bindr.gdcb.iastate.edu:8080/ZiFDB/controller/searchFinger?target=GTT) | - | CoDA | | F2 | RQDNLGR | [GAG](http://bindr.gdcb.iastate.edu:8080/ZiFDB/controller/searchFinger?target=GAG) | - | CoDA | | F3 | QGGNLTL | [TAA](http://bindr.gdcb.iastate.edu:8080/ZiFDB/controller/searchFinger?target=TAA) | - | CoDA |   [ZF DNA Sequence](javascript:CoDAPopupArrayWindow("ZFA-unknown-37","TTTVLAR","RQDNLGR","QGGNLTL")) | |
| --- | --- | --- | --- | --- | --- | --- | --- | --- | --- | --- | --- | --- | --- | --- | --- | --- | --- | --- | --- | --- | --- | --- | --- | --- |
| [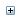](javascript:TreeView_ToggleNode(ctl00_ContentPlaceHolder1_tree12_Data,74,document.getElementById('ctl00_ContentPlaceHolder1_tree12n74'),'%20',document.getElementById('ctl00_ContentPlaceHolder1_tree12n74Nodes'))) | | ZFA-unknown-38 1233 t[GTAGCTGCT](http://bindr.gdcb.iastate.edu:8080/ZiFDB/controller/searchArray?site=GCTGCTGTA)a 1223  1233 aCATCGACGAt 1223 | |  |

|  | 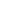 | | | FINGER | HELIX | TRIPLET | REFERENCE NUMBER | SOURCE | | --- | --- | --- | --- | --- | | F1 | MKNTLTR | [GCT](http://bindr.gdcb.iastate.edu:8080/ZiFDB/controller/searchFinger?target=GCT) | - | CoDA | | F2 | QRSDLTR | [GCT](http://bindr.gdcb.iastate.edu:8080/ZiFDB/controller/searchFinger?target=GCT) | - | CoDA | | F3 | QSGTLTR | [GTA](http://bindr.gdcb.iastate.edu:8080/ZiFDB/controller/searchFinger?target=GTA) | - | CoDA |   [ZF DNA Sequence](javascript:CoDAPopupArrayWindow("ZFA-unknown-38","MKNTLTR","QRSDLTR","QSGTLTR")) | |
| --- | --- | --- | --- | --- | --- | --- | --- | --- | --- | --- | --- | --- | --- | --- | --- | --- | --- | --- | --- | --- | --- | --- | --- | --- |
| [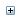](javascript:TreeView_ToggleNode(ctl00_ContentPlaceHolder1_tree12_Data,76,document.getElementById('ctl00_ContentPlaceHolder1_tree12n76'),'%20',document.getElementById('ctl00_ContentPlaceHolder1_tree12n76Nodes'))) | | ZFA-unknown-39 1244 g[GTAGCTGGA](http://bindr.gdcb.iastate.edu:8080/ZiFDB/controller/searchArray?site=GGAGCTGTA)t 1234  1244 cCATCGACCTa 1234 | |  |

|  | 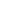 | | | FINGER | HELIX | TRIPLET | REFERENCE NUMBER | SOURCE | | --- | --- | --- | --- | --- | | F1 | RPAKLVL | [GGA](http://bindr.gdcb.iastate.edu:8080/ZiFDB/controller/searchFinger?target=GGA) | - | CoDA | | F2 | QRSDLTR | [GCT](http://bindr.gdcb.iastate.edu:8080/ZiFDB/controller/searchFinger?target=GCT) | - | CoDA | | F3 | QSGTLTR | [GTA](http://bindr.gdcb.iastate.edu:8080/ZiFDB/controller/searchFinger?target=GTA) | - | CoDA |   [ZF DNA Sequence](javascript:CoDAPopupArrayWindow("ZFA-unknown-39","RPAKLVL","QRSDLTR","QSGTLTR")) | |
| --- | --- | --- | --- | --- | --- | --- | --- | --- | --- | --- | --- | --- | --- | --- | --- | --- | --- | --- | --- | --- | --- | --- | --- | --- |
| [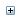](javascript:TreeView_ToggleNode(ctl00_ContentPlaceHolder1_tree12_Data,78,document.getElementById('ctl00_ContentPlaceHolder1_tree12n78'),'%20',document.getElementById('ctl00_ContentPlaceHolder1_tree12n78Nodes'))) | | ZFA-unknown-40 1332 a[TAAGAGGTA](http://bindr.gdcb.iastate.edu:8080/ZiFDB/controller/searchArray?site=GTAGAGTAA)t 1322  1332 tATTCTCCATa 1322 | |  |

|  | 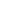 | | | FINGER | HELIX | TRIPLET | REFERENCE NUMBER | SOURCE | | --- | --- | --- | --- | --- | | F1 | QQQALVR | [GTA](http://bindr.gdcb.iastate.edu:8080/ZiFDB/controller/searchFinger?target=GTA) | - | CoDA | | F2 | RQDNLGR | [GAG](http://bindr.gdcb.iastate.edu:8080/ZiFDB/controller/searchFinger?target=GAG) | - | CoDA | | F3 | QGGNLTL | [TAA](http://bindr.gdcb.iastate.edu:8080/ZiFDB/controller/searchFinger?target=TAA) | - | CoDA |   [ZF DNA Sequence](javascript:CoDAPopupArrayWindow("ZFA-unknown-40","QQQALVR","RQDNLGR","QGGNLTL")) | |
| --- | --- | --- | --- | --- | --- | --- | --- | --- | --- | --- | --- | --- | --- | --- | --- | --- | --- | --- | --- | --- | --- | --- | --- | --- |
| [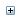](javascript:TreeView_ToggleNode(ctl00_ContentPlaceHolder1_tree12_Data,80,document.getElementById('ctl00_ContentPlaceHolder1_tree12n80'),'%20',document.getElementById('ctl00_ContentPlaceHolder1_tree12n80Nodes'))) | | ZFA-unknown-41 1340 g[GAGGAAGAT](http://bindr.gdcb.iastate.edu:8080/ZiFDB/controller/searchArray?site=GATGAAGAG)a 1330  1340 cCTCCTTCTAt 1330 | |  |

|  | 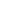 | | | FINGER | HELIX | TRIPLET | REFERENCE NUMBER | SOURCE | | --- | --- | --- | --- | --- | | F1 | TSQMLVV | [GAT](http://bindr.gdcb.iastate.edu:8080/ZiFDB/controller/searchFinger?target=GAT) | - | CoDA | | F2 | QQTNLTR | [GAA](http://bindr.gdcb.iastate.edu:8080/ZiFDB/controller/searchFinger?target=GAA) | - | CoDA | | F3 | RRDNLNR | [GAG](http://bindr.gdcb.iastate.edu:8080/ZiFDB/controller/searchFinger?target=GAG) | - | CoDA |   [ZF DNA Sequence](javascript:CoDAPopupArrayWindow("ZFA-unknown-41","TSQMLVV","QQTNLTR","RRDNLNR")) | |
| --- | --- | --- | --- | --- | --- | --- | --- | --- | --- | --- | --- | --- | --- | --- | --- | --- | --- | --- | --- | --- | --- | --- | --- | --- |
| [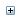](javascript:TreeView_ToggleNode(ctl00_ContentPlaceHolder1_tree12_Data,82,document.getElementById('ctl00_ContentPlaceHolder1_tree12n82'),'%20',document.getElementById('ctl00_ContentPlaceHolder1_tree12n82Nodes'))) | | ZFA-unknown-42 1343 g[TGGGAGGAA](http://bindr.gdcb.iastate.edu:8080/ZiFDB/controller/searchArray?site=GAAGAGTGG)g 1333  1343 cACCCTCCTTc 1333 | |  |

|  | 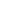 | | | FINGER | HELIX | TRIPLET | REFERENCE NUMBER | SOURCE | | --- | --- | --- | --- | --- | | F1 | QASNLLR | [GAA](http://bindr.gdcb.iastate.edu:8080/ZiFDB/controller/searchFinger?target=GAA) | - | CoDA | | F2 | RQDNLGR | [GAG](http://bindr.gdcb.iastate.edu:8080/ZiFDB/controller/searchFinger?target=GAG) | - | CoDA | | F3 | RMDHLAG | [TGG](http://bindr.gdcb.iastate.edu:8080/ZiFDB/controller/searchFinger?target=TGG) | - | CoDA |   [ZF DNA Sequence](javascript:CoDAPopupArrayWindow("ZFA-unknown-42","QASNLLR","RQDNLGR","RMDHLAG")) | |
| --- | --- | --- | --- | --- | --- | --- | --- | --- | --- | --- | --- | --- | --- | --- | --- | --- | --- | --- | --- | --- | --- | --- | --- | --- |
| [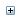](javascript:TreeView_ToggleNode(ctl00_ContentPlaceHolder1_tree12_Data,84,document.getElementById('ctl00_ContentPlaceHolder1_tree12n84'),'%20',document.getElementById('ctl00_ContentPlaceHolder1_tree12n84Nodes'))) | | ZFA-unknown-43 1344 t[GTGGGAGGA](http://bindr.gdcb.iastate.edu:8080/ZiFDB/controller/searchArray?site=GGAGGAGTG)a 1334  1344 aCACCCTCCTt 1334 | |  |

|  | 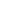 | | | FINGER | HELIX | TRIPLET | REFERENCE NUMBER | SOURCE | | --- | --- | --- | --- | --- | | F1 | RTDRLIR | [GGA](http://bindr.gdcb.iastate.edu:8080/ZiFDB/controller/searchFinger?target=GGA) | - | CoDA | | F2 | QSAHLKR | [GGA](http://bindr.gdcb.iastate.edu:8080/ZiFDB/controller/searchFinger?target=GGA) | - | CoDA | | F3 | RNTALQH | [GTG](http://bindr.gdcb.iastate.edu:8080/ZiFDB/controller/searchFinger?target=GTG) | - | CoDA |   [ZF DNA Sequence](javascript:CoDAPopupArrayWindow("ZFA-unknown-43","RTDRLIR","QSAHLKR","RNTALQH")) | |
| --- | --- | --- | --- | --- | --- | --- | --- | --- | --- | --- | --- | --- | --- | --- | --- | --- | --- | --- | --- | --- | --- | --- | --- | --- |
| [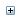](javascript:TreeView_ToggleNode(ctl00_ContentPlaceHolder1_tree12_Data,86,document.getElementById('ctl00_ContentPlaceHolder1_tree12n86'),'%20',document.getElementById('ctl00_ContentPlaceHolder1_tree12n86Nodes'))) | | ZFA-unknown-44 1347 a[GCTGTGGGA](http://bindr.gdcb.iastate.edu:8080/ZiFDB/controller/searchArray?site=GGAGTGGCT)g 1337  1347 tCGACACCCTc 1337 | |  |

|  | 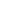 | | | FINGER | HELIX | TRIPLET | REFERENCE NUMBER | SOURCE | | --- | --- | --- | --- | --- | | F1 | THAHLTR | [GGA](http://bindr.gdcb.iastate.edu:8080/ZiFDB/controller/searchFinger?target=GGA) | - | CoDA | | F2 | RREVLEN | [GTG](http://bindr.gdcb.iastate.edu:8080/ZiFDB/controller/searchFinger?target=GTG) | - | CoDA | | F3 | VGASLKR | [GCT](http://bindr.gdcb.iastate.edu:8080/ZiFDB/controller/searchFinger?target=GCT) | - | CoDA |   [ZF DNA Sequence](javascript:CoDAPopupArrayWindow("ZFA-unknown-44","THAHLTR","RREVLEN","VGASLKR")) | |
| --- | --- | --- | --- | --- | --- | --- | --- | --- | --- | --- | --- | --- | --- | --- | --- | --- | --- | --- | --- | --- | --- | --- | --- | --- |
| [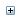](javascript:TreeView_ToggleNode(ctl00_ContentPlaceHolder1_tree12_Data,88,document.getElementById('ctl00_ContentPlaceHolder1_tree12n88'),'%20',document.getElementById('ctl00_ContentPlaceHolder1_tree12n88Nodes'))) | | ZFA-unknown-45 1349 c[TGGGCAACG](http://bindr.gdcb.iastate.edu:8080/ZiFDB/controller/searchArray?site=ACGGCATGG)t 1359  1349 gACCCGTTGCa 1359 | |  |

|  | 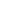 | | | FINGER | HELIX | TRIPLET | REFERENCE NUMBER | SOURCE | | --- | --- | --- | --- | --- | | F1 | RSQTLAQ | [ACG](http://bindr.gdcb.iastate.edu:8080/ZiFDB/controller/searchFinger?target=ACG) | - | CoDA | | F2 | QSTTLKR | [GCA](http://bindr.gdcb.iastate.edu:8080/ZiFDB/controller/searchFinger?target=GCA) | - | CoDA | | F3 | RSDHLSL | [TGG](http://bindr.gdcb.iastate.edu:8080/ZiFDB/controller/searchFinger?target=TGG) | - | CoDA |   [ZF DNA Sequence](javascript:CoDAPopupArrayWindow("ZFA-unknown-45","RSQTLAQ","QSTTLKR","RSDHLSL")) | |
| --- | --- | --- | --- | --- | --- | --- | --- | --- | --- | --- | --- | --- | --- | --- | --- | --- | --- | --- | --- | --- | --- | --- | --- | --- |
| [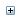](javascript:TreeView_ToggleNode(ctl00_ContentPlaceHolder1_tree12_Data,90,document.getElementById('ctl00_ContentPlaceHolder1_tree12n90'),'%20',document.getElementById('ctl00_ContentPlaceHolder1_tree12n90Nodes'))) | | ZFA-unknown-46 1368 g[TGTGCTGGC](http://bindr.gdcb.iastate.edu:8080/ZiFDB/controller/searchArray?site=GGCGCTTGT)c 1378  1368 cACACGACCGg 1378 | |  |

|  | 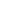 | | | FINGER | HELIX | TRIPLET | REFERENCE NUMBER | SOURCE | | --- | --- | --- | --- | --- | | F1 | APSKLAR | [GGC](http://bindr.gdcb.iastate.edu:8080/ZiFDB/controller/searchFinger?target=GGC) | - | CoDA | | F2 | QRSDLTR | [GCT](http://bindr.gdcb.iastate.edu:8080/ZiFDB/controller/searchFinger?target=GCT) | - | CoDA | | F3 | QPHGLRH | [TGT](http://bindr.gdcb.iastate.edu:8080/ZiFDB/controller/searchFinger?target=TGT) | - | CoDA |   [ZF DNA Sequence](javascript:CoDAPopupArrayWindow("ZFA-unknown-46","APSKLAR","QRSDLTR","QPHGLRH")) | |
| --- | --- | --- | --- | --- | --- | --- | --- | --- | --- | --- | --- | --- | --- | --- | --- | --- | --- | --- | --- | --- | --- | --- | --- | --- |
| [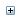](javascript:TreeView_ToggleNode(ctl00_ContentPlaceHolder1_tree12_Data,92,document.getElementById('ctl00_ContentPlaceHolder1_tree12n92'),'%20',document.getElementById('ctl00_ContentPlaceHolder1_tree12n92Nodes'))) | | ZFA-unknown-47 1406 g[TGGGGTGAA](http://bindr.gdcb.iastate.edu:8080/ZiFDB/controller/searchArray?site=GAAGGTTGG)t 1396  1406 cACCCCACTTa 1396 | |  |

|  | 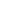 | | | FINGER | HELIX | TRIPLET | REFERENCE NUMBER | SOURCE | | --- | --- | --- | --- | --- | | F1 | RKPNLLR | [GAA](http://bindr.gdcb.iastate.edu:8080/ZiFDB/controller/searchFinger?target=GAA) | - | CoDA | | F2 | EAHHLSR | [GGT](http://bindr.gdcb.iastate.edu:8080/ZiFDB/controller/searchFinger?target=GGT) | - | CoDA | | F3 | RSDHLSL | [TGG](http://bindr.gdcb.iastate.edu:8080/ZiFDB/controller/searchFinger?target=TGG) | - | CoDA |   [ZF DNA Sequence](javascript:CoDAPopupArrayWindow("ZFA-unknown-47","RKPNLLR","EAHHLSR","RSDHLSL")) | |
| --- | --- | --- | --- | --- | --- | --- | --- | --- | --- | --- | --- | --- | --- | --- | --- | --- | --- | --- | --- | --- | --- | --- | --- | --- |
| [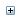](javascript:TreeView_ToggleNode(ctl00_ContentPlaceHolder1_tree12_Data,94,document.getElementById('ctl00_ContentPlaceHolder1_tree12n94'),'%20',document.getElementById('ctl00_ContentPlaceHolder1_tree12n94Nodes'))) | | ZFA-unknown-48 1408 t[GGTGGGGTG](http://bindr.gdcb.iastate.edu:8080/ZiFDB/controller/searchArray?site=GTGGGGGGT)a 1398  1408 aCCACCCCACt 1398 | |  |

|  | 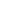 | | | FINGER | HELIX | TRIPLET | REFERENCE NUMBER | SOURCE | | --- | --- | --- | --- | --- | | F1 | SRFTLGR | [GTG](http://bindr.gdcb.iastate.edu:8080/ZiFDB/controller/searchFinger?target=GTG) | - | CoDA | | F2 | RREHLVR | [GGG](http://bindr.gdcb.iastate.edu:8080/ZiFDB/controller/searchFinger?target=GGG) | - | CoDA | | F3 | VDHHLRR | [GGT](http://bindr.gdcb.iastate.edu:8080/ZiFDB/controller/searchFinger?target=GGT) | - | CoDA |   [ZF DNA Sequence](javascript:CoDAPopupArrayWindow("ZFA-unknown-48","SRFTLGR","RREHLVR","VDHHLRR")) | |
| --- | --- | --- | --- | --- | --- | --- | --- | --- | --- | --- | --- | --- | --- | --- | --- | --- | --- | --- | --- | --- | --- | --- | --- | --- |
| [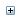](javascript:TreeView_ToggleNode(ctl00_ContentPlaceHolder1_tree12_Data,96,document.getElementById('ctl00_ContentPlaceHolder1_tree12n96'),'%20',document.getElementById('ctl00_ContentPlaceHolder1_tree12n96Nodes'))) | | ZFA-unknown-49 1420 g[GCAGCCTGC](http://bindr.gdcb.iastate.edu:8080/ZiFDB/controller/searchArray?site=TGCGCCGCA)a 1410  1420 cCGTCGGACGt 1410 | |  |

|  | 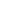 | | | FINGER | HELIX | TRIPLET | REFERENCE NUMBER | SOURCE | | --- | --- | --- | --- | --- | | F1 | RGRNLEM | [TGC](http://bindr.gdcb.iastate.edu:8080/ZiFDB/controller/searchFinger?target=TGC) | - | CoDA | | F2 | DSSVLRR | [GCC](http://bindr.gdcb.iastate.edu:8080/ZiFDB/controller/searchFinger?target=GCC) | - | CoDA | | F3 | QGGTLRR | [GCA](http://bindr.gdcb.iastate.edu:8080/ZiFDB/controller/searchFinger?target=GCA) | - | CoDA |   [ZF DNA Sequence](javascript:CoDAPopupArrayWindow("ZFA-unknown-49","RGRNLEM","DSSVLRR","QGGTLRR")) | |
| --- | --- | --- | --- | --- | --- | --- | --- | --- | --- | --- | --- | --- | --- | --- | --- | --- | --- | --- | --- | --- | --- | --- | --- | --- |
| [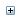](javascript:TreeView_ToggleNode(ctl00_ContentPlaceHolder1_tree12_Data,98,document.getElementById('ctl00_ContentPlaceHolder1_tree12n98'),'%20',document.getElementById('ctl00_ContentPlaceHolder1_tree12n98Nodes'))) | | ZFA-unknown-50 1423 a[TAGGCAGCC](http://bindr.gdcb.iastate.edu:8080/ZiFDB/controller/searchArray?site=GCCGCATAG)t 1413  1423 tATCCGTCGGa 1413 | |  |

|  |  | | | FINGER | HELIX | TRIPLET | REFERENCE NUMBER | SOURCE | | --- | --- | --- | --- | --- | | F1 | DPSTLRR | [GCC](http://bindr.gdcb.iastate.edu:8080/ZiFDB/controller/searchFinger?target=GCC) | - | CoDA | | F2 | QSTTLKR | [GCA](http://bindr.gdcb.iastate.edu:8080/ZiFDB/controller/searchFinger?target=GCA) | - | CoDA | | F3 | RRDGLAG | [TAG](http://bindr.gdcb.iastate.edu:8080/ZiFDB/controller/searchFinger?target=TAG) | - | CoDA |   [ZF DNA Sequence](javascript:CoDAPopupArrayWindow("ZFA-unknown-50","DPSTLRR","QSTTLKR","RRDGLAG")) | |
| --- | --- | --- | --- | --- | --- | --- | --- | --- | --- | --- | --- | --- | --- | --- | --- | --- | --- | --- | --- | --- | --- | --- | --- | --- |
|  | | ZFA-unknown-51 1429 a[GTGGTGGCT](http://bindr.gdcb.iastate.edu:8080/ZiFDB/controller/searchArray?site=GCTGTGGTG)g 1439  1429 tCACCACCGAc 1439 | |  |

|  |  | | | FINGER | HELIX | TRIPLET | REFERENCE NUMBER | SOURCE | | --- | --- | --- | --- | --- | | F1 | TKPILVR | [GCT](http://bindr.gdcb.iastate.edu:8080/ZiFDB/controller/searchFinger?target=GCT) | - | CoDA | | F2 | RREVLEN | [GTG](http://bindr.gdcb.iastate.edu:8080/ZiFDB/controller/searchFinger?target=GTG) | - | CoDA | | F3 | RKDALHV | [GTG](http://bindr.gdcb.iastate.edu:8080/ZiFDB/controller/searchFinger?target=GTG) | - | CoDA |   [ZF DNA Sequence](javascript:CoDAPopupArrayWindow("ZFA-unknown-51","TKPILVR","RREVLEN","RKDALHV")) | |
| --- | --- | --- | --- | --- | --- | --- | --- | --- | --- | --- | --- | --- | --- | --- | --- | --- | --- | --- | --- | --- | --- | --- | --- | --- |
|  | | ZFA-unknown-52 1432 g[GTGGCTGGT](http://bindr.gdcb.iastate.edu:8080/ZiFDB/controller/searchArray?site=GGTGCTGTG)g 1442  1432 cCACCGACCAc 1442 | |  |

|  |  | | | FINGER | HELIX | TRIPLET | REFERENCE NUMBER | SOURCE | | --- | --- | --- | --- | --- | | F1 | RRQKLTI | [GGT](http://bindr.gdcb.iastate.edu:8080/ZiFDB/controller/searchFinger?target=GGT) | - | CoDA | | F2 | QRSDLTR | [GCT](http://bindr.gdcb.iastate.edu:8080/ZiFDB/controller/searchFinger?target=GCT) | - | CoDA | | F3 | RPDALPR | [GTG](http://bindr.gdcb.iastate.edu:8080/ZiFDB/controller/searchFinger?target=GTG) | - | CoDA |   [ZF DNA Sequence](javascript:CoDAPopupArrayWindow("ZFA-unknown-52","RRQKLTI","QRSDLTR","RPDALPR")) | |
| --- | --- | --- | --- | --- | --- | --- | --- | --- | --- | --- | --- | --- | --- | --- | --- | --- | --- | --- | --- | --- | --- | --- | --- | --- |
|  | | ZFA-unknown-53 1435 g[GCTGGTGTG](http://bindr.gdcb.iastate.edu:8080/ZiFDB/controller/searchArray?site=GTGGGTGCT)g 1445  1435 cCGACCACACc 1445 | |  |

|  |  | | | FINGER | HELIX | TRIPLET | REFERENCE NUMBER | SOURCE | | --- | --- | --- | --- | --- | | F1 | RRFILSR | [GTG](http://bindr.gdcb.iastate.edu:8080/ZiFDB/controller/searchFinger?target=GTG) | - | CoDA | | F2 | EAHHLSR | [GGT](http://bindr.gdcb.iastate.edu:8080/ZiFDB/controller/searchFinger?target=GGT) | - | CoDA | | F3 | EGSGLKR | [GCT](http://bindr.gdcb.iastate.edu:8080/ZiFDB/controller/searchFinger?target=GCT) | - | CoDA |   [ZF DNA Sequence](javascript:CoDAPopupArrayWindow("ZFA-unknown-53","RRFILSR","EAHHLSR","EGSGLKR")) | |
| --- | --- | --- | --- | --- | --- | --- | --- | --- | --- | --- | --- | --- | --- | --- | --- | --- | --- | --- | --- | --- | --- | --- | --- | --- |
|  | | ZFA-unknown-54 1438 t[GGTGTGGCT](http://bindr.gdcb.iastate.edu:8080/ZiFDB/controller/searchArray?site=GCTGTGGGT)a 1448  1438 aCCACACCGAt 1448 | |  |

|  |  | | | FINGER | HELIX | TRIPLET | REFERENCE NUMBER | SOURCE | | --- | --- | --- | --- | --- | | F1 | TKPILVR | [GCT](http://bindr.gdcb.iastate.edu:8080/ZiFDB/controller/searchFinger?target=GCT) | - | CoDA | | F2 | RREVLEN | [GTG](http://bindr.gdcb.iastate.edu:8080/ZiFDB/controller/searchFinger?target=GTG) | - | CoDA | | F3 | VKHGLTR | [GGT](http://bindr.gdcb.iastate.edu:8080/ZiFDB/controller/searchFinger?target=GGT) | - | CoDA |   [ZF DNA Sequence](javascript:CoDAPopupArrayWindow("ZFA-unknown-54","TKPILVR","RREVLEN","VKHGLTR")) | |
| --- | --- | --- | --- | --- | --- | --- | --- | --- | --- | --- | --- | --- | --- | --- | --- | --- | --- | --- | --- | --- | --- | --- | --- | --- |
|  | | ZFA-unknown-55 1525 c[TTAGGGAAC](http://bindr.gdcb.iastate.edu:8080/ZiFDB/controller/searchArray?site=AACGGGTTA)a 1515  1525 gAATCCCTTGt 1515 | |  |

|  |  | | | FINGER | HELIX | TRIPLET | REFERENCE NUMBER | SOURCE | | --- | --- | --- | --- | --- | | F1 | HRTNLIA | [AAC](http://bindr.gdcb.iastate.edu:8080/ZiFDB/controller/searchFinger?target=AAC) | - | CoDA | | F2 | RREHLVR | [GGG](http://bindr.gdcb.iastate.edu:8080/ZiFDB/controller/searchFinger?target=GGG) | - | CoDA | | F3 | QQTGLNV | [TTA](http://bindr.gdcb.iastate.edu:8080/ZiFDB/controller/searchFinger?target=TTA) | - | CoDA |   [ZF DNA Sequence](javascript:CoDAPopupArrayWindow("ZFA-unknown-55","HRTNLIA","RREHLVR","QQTGLNV")) | |
| --- | --- | --- | --- | --- | --- | --- | --- | --- | --- | --- | --- | --- | --- | --- | --- | --- | --- | --- | --- | --- | --- | --- | --- | --- |
|  | | ZFA-unknown-56 1535 a[GTAGTTGGA](http://bindr.gdcb.iastate.edu:8080/ZiFDB/controller/searchArray?site=GGAGTTGTA)c 1525  1535 tCATCAACCTg 1525 | |  |

|  |  | | | FINGER | HELIX | TRIPLET | REFERENCE NUMBER | SOURCE | | --- | --- | --- | --- | --- | | F1 | RSTHLRV | [GGA](http://bindr.gdcb.iastate.edu:8080/ZiFDB/controller/searchFinger?target=GGA) | - | CoDA | | F2 | HKSSLTR | [GTT](http://bindr.gdcb.iastate.edu:8080/ZiFDB/controller/searchFinger?target=GTT) | - | CoDA | | F3 | QSTSLQR | [GTA](http://bindr.gdcb.iastate.edu:8080/ZiFDB/controller/searchFinger?target=GTA) | - | CoDA |   [ZF DNA Sequence](javascript:CoDAPopupArrayWindow("ZFA-unknown-56","RSTHLRV","HKSSLTR","QSTSLQR")) | |
| --- | --- | --- | --- | --- | --- | --- | --- | --- | --- | --- | --- | --- | --- | --- | --- | --- | --- | --- | --- | --- | --- | --- | --- | --- |
|  | | ZFA-unknown-57 1583 t[TAGGCAGAA](http://bindr.gdcb.iastate.edu:8080/ZiFDB/controller/searchArray?site=GAAGCATAG)t 1573  1583 aATCCGTCTTa 1573 | |  |
