# Supplementary material for: Zinc finger nucleases for targeted mutagenesis and repair of the sickle-cell disease mutation: An in-silico study
Source: BMC Blood Disord. 2012 May 14;12:5. doi: 10.1186/1471-2326-12-5 (PMC3407482; doi:10.1186/1471-2326-12-5)
Supplement: Additional file 3 — A detailed list of thefourZFNs specific to the Hemoglobin, beta gene-sequences. This file lists the four ZFNs that specifically bind and cleave sequences within the Hemoglobin, beta gene [file 1471-2326-12-5-S3.doc]

**Zinc Finger Site Type:** Nuclease
**Zinc Finger Engineering Method:** CoDA
**Sequence Name** : unknown
**Sequence Length**:1608
**Nucleotide Sequence** :nACATTTGCTTCTGACACAACTGTGTTCACTAGCAACCTCAAACAGACACCATGGTGCATCTGACTCCTGAGGAGAAGTCTGCCGTTACTGCCCTGTGGGGCAAGGTGAACGTGGATGAAGTTGGTGGTGAGGCCCTGGGCAGGTTGGTATCAAGGTTACAAGACAGGTTTAAGGAGACCAATAGAAACTGGGCATGTGGAGACAGAGAAGACTCTTGGGTTTCTGATAGGCACTGACTCTCTCTGCCTATTGGTCTATTTTCCCACCCTTAGGCTGCTGGTGGTCTACCCTTGGACCCAGAGGTTCTTTGAGTCCTTTGGGGATCTGTCCACTCCTGATGCTGTTATGGGCAACCCTAAGGTGAAGGCTCATGGCAAGAAAGTGCTCGGTGCCTTTAGTGATGGCCTGGCTCACCTGGACAACCTCAAGGGCACCTTTGCCACACTGAGTGAGCTGCACTGTGACAAGCTGCACGTGGATCCTGAGAACTTCAGGGTGAGTCTATGGGACGCTTGATGTTTTCTTTCCCCTTCTTTTCTATGGTTAAGTTCATGTCATAGGAAGGGGATAAGTAACAGGGTACAGTTTAGAATGGGAAACAGACGAATGATTGCATCAGTGTGGAAGTCTCAGGATCGTTTTAGTTTCTTTTATTTGCTGTTCATAACAATTGTTTTCTTTTGTTTAATTCTTGCTTTCTTTTTTTTTCTTCTCCGCAATTTTTACTATTATACTTAATGCCTTAACATTGTGTATAACAAAAGGAAATATCTCTGAGATACATTAAGTAACTTAAAAAAAAACTTTACACAGTCTGCCTAGTACATTACTATTTGGAATATATGTGTGCTTATTTGCATATTCATAATCTCCCTACTTTATTTTCTTTTATTTTTAATTGATACATAATCATTATACATATTTATGGGTTAAAGTGTAATGTTTTAATATGTGTACACATATTGACCAAATCAGGGTAATTTTGCATTTGTAATTTTAAAAAATGCTTTCTTCTTTTAATATACTTTTTTGTTTATCTTATTTCTAATACTTTCCCTAATCTCTTTCTTTCAGGGCAATAATGATACAATGTATCATGCCTCTTTGCACCATTCTAAAGAATAACAGTGATAATTTCTGGGTTAAGGCAATAGCAATATCTCTGCATATAAATATTTCTGCATATAAATTGTAACTGATGTAAGAGGTTTCATATTGCTAATAGCAGCTACAATCCAGCTACCATTCTGCTTTTATTTTATGGTTGGGATAAGGCTGGATTATTCTGAGTCCAAGCTAGGCCCTTTTGCTAATCATGTTCATACCTCTTATCTTCCTCCCACAGCTCCTGGGCAACGTGCTGGTCTGTGTGCTGGCCCATCACTTTGGCAAAGAATTCACCCCACCAGTGCAGGCTGCCTATCAGAAAGTGGTGGCTGGTGTGGCTAATGCCCTGGCCCACAAGTATCACTAAGCTCGCTTTCTTGCTGTCCAATTTCTATTAAAGGTTCCTTTGTTCCCTAAGTCCAACTACTAAACTGGGGGATATTATGAAGGGCCTTGAGCATCTGGATTCTGCCTAATAAAAAACATTTATTTTCATTGCn
**Selected Module Sets:**
**Left Module Count:** 3
**Spacer Nucleotide Count:** 5,6,7
**Right Module Count:** 3
**Ignore Asp Overlap:**False

The results below are zinc finger *nucleases* that can be constructed using CoDA. Note that other methods (including modular assembly and OPEN) can also potentially be used to target the input sequence of interest.”

**Sort By: Hide intron splice sites**

**[

](http://zifit.partners.org/ZiFiT/CoDAZiFiTNuclease.aspx#ctl00_ContentPlaceHolder1_tree12_SkipLink)**

| [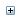](javascript:TreeView_ToggleNode(ctl00_ContentPlaceHolder1_tree12_Data,0,document.getElementById('ctl00_ContentPlaceHolder1_tree12n0'),'%20',document.getElementById('ctl00_ContentPlaceHolder1_tree12n0Nodes'))) | ZFN-unknown-SP-5-1 82 cCGTTACTGCCCTGT[GGGGCAAGG](http://bindr.gdcb.iastate.edu:8080/ZiFDB/controller/searchArray?site=AGGGCAGGG)t 106  82 g[GCAATGACG](http://bindr.gdcb.iastate.edu:8080/ZiFDB/controller/searchArray?site=ACGGTAGCA)GGACACCCCGTTCCa 106 |
| --- | --- |

|  | 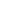 | | | FINGER | HELIX | TRIPLET | REFERENCE NUMBER | SOURCE | | --- | --- | --- | --- | --- | | Left F1 | RNITLVR | [ACG](http://bindr.gdcb.iastate.edu:8080/ZiFDB/controller/searchFinger?target=ACG) | - | CoDA | | Left F2 | QRSSLVR | [GTA](http://bindr.gdcb.iastate.edu:8080/ZiFDB/controller/searchFinger?target=GTA) | - | CoDA | | Left F3 | QDNTLRR | [GCA](http://bindr.gdcb.iastate.edu:8080/ZiFDB/controller/searchFinger?target=GCA) | - | CoDA | | Right F1 | RNEHLKV | [AGG](http://bindr.gdcb.iastate.edu:8080/ZiFDB/controller/searchFinger?target=AGG) | - | CoDA | | Right F2 | QSTTLKR | [GCA](http://bindr.gdcb.iastate.edu:8080/ZiFDB/controller/searchFinger?target=GCA) | - | CoDA | | Right F3 | RTEHLAR | [GGG](http://bindr.gdcb.iastate.edu:8080/ZiFDB/controller/searchFinger?target=GGG) | - | CoDA |   [ZF DNA Sequence](javascript:CoDAPopupNucleaseWindow("Left-ZFN-unknown-SP-5-1","RNITLVR","QRSSLVR","QDNTLRR","Right-ZFN-unknown-SP-5-1","RNEHLKV","QSTTLKR","RTEHLAR")) | |
| --- | --- | --- | --- | --- | --- | --- | --- | --- | --- | --- | --- | --- | --- | --- | --- | --- | --- | --- | --- | --- | --- | --- | --- | --- | --- | --- | --- | --- | --- | --- | --- | --- | --- | --- | --- | --- | --- | --- | --- |
| [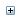](javascript:TreeView_ToggleNode(ctl00_ContentPlaceHolder1_tree12_Data,2,document.getElementById('ctl00_ContentPlaceHolder1_tree12n2'),'%20',document.getElementById('ctl00_ContentPlaceHolder1_tree12n2Nodes'))) | | ZFN-unknown-SP-7-1 1333 cTTCCTCCCACAGCTCC[TGGGCAACG](http://bindr.gdcb.iastate.edu:8080/ZiFDB/controller/searchArray?site=ACGGCATGG)t 1359  1333 g[AAGGAGGGT](http://bindr.gdcb.iastate.edu:8080/ZiFDB/controller/searchArray?site=GAAGAGTGG)GTCGAGGACCCGTTGCa 1359 | |  |

|  | 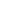 | | | FINGER | HELIX | TRIPLET | REFERENCE NUMBER | SOURCE | | --- | --- | --- | --- | --- | | Left F1 | QASNLLR | [GAA](http://bindr.gdcb.iastate.edu:8080/ZiFDB/controller/searchFinger?target=GAA) | - | CoDA | | Left F2 | RQDNLGR | [GAG](http://bindr.gdcb.iastate.edu:8080/ZiFDB/controller/searchFinger?target=GAG) | - | CoDA | | Left F3 | RMDHLAG | [TGG](http://bindr.gdcb.iastate.edu:8080/ZiFDB/controller/searchFinger?target=TGG) | - | CoDA | | Right F1 | RSQTLAQ | [ACG](http://bindr.gdcb.iastate.edu:8080/ZiFDB/controller/searchFinger?target=ACG) | - | CoDA | | Right F2 | QSTTLKR | [GCA](http://bindr.gdcb.iastate.edu:8080/ZiFDB/controller/searchFinger?target=GCA) | - | CoDA | | Right F3 | RSDHLSL | [TGG](http://bindr.gdcb.iastate.edu:8080/ZiFDB/controller/searchFinger?target=TGG) | - | CoDA |   [ZF DNA Sequence](javascript:CoDAPopupNucleaseWindow("Left-ZFN-unknown-SP-7-1","QASNLLR","RQDNLGR","RMDHLAG","Right-ZFN-unknown-SP-7-1","RSQTLAQ","QSTTLKR","RSDHLSL")) | |
| --- | --- | --- | --- | --- | --- | --- | --- | --- | --- | --- | --- | --- | --- | --- | --- | --- | --- | --- | --- | --- | --- | --- | --- | --- | --- | --- | --- | --- | --- | --- | --- | --- | --- | --- | --- | --- | --- | --- | --- |
| [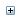](javascript:TreeView_ToggleNode(ctl00_ContentPlaceHolder1_tree12_Data,4,document.getElementById('ctl00_ContentPlaceHolder1_tree12n4'),'%20',document.getElementById('ctl00_ContentPlaceHolder1_tree12n4Nodes'))) | | ZFN-unknown-SP-6-1 1334 tTCCTCCCACAGCTCC[TGGGCAACG](http://bindr.gdcb.iastate.edu:8080/ZiFDB/controller/searchArray?site=ACGGCATGG)t 1359  1334 a[AGGAGGGTG](http://bindr.gdcb.iastate.edu:8080/ZiFDB/controller/searchArray?site=GGAGGAGTG)TCGAGGACCCGTTGCa 1359 | |  |

|  | 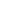 | | | FINGER | HELIX | TRIPLET | REFERENCE NUMBER | SOURCE | | --- | --- | --- | --- | --- | | Left F1 | RTDRLIR | [GGA](http://bindr.gdcb.iastate.edu:8080/ZiFDB/controller/searchFinger?target=GGA) | - | CoDA | | Left F2 | QSAHLKR | [GGA](http://bindr.gdcb.iastate.edu:8080/ZiFDB/controller/searchFinger?target=GGA) | - | CoDA | | Left F3 | RNTALQH | [GTG](http://bindr.gdcb.iastate.edu:8080/ZiFDB/controller/searchFinger?target=GTG) | - | CoDA | | Right F1 | RSQTLAQ | [ACG](http://bindr.gdcb.iastate.edu:8080/ZiFDB/controller/searchFinger?target=ACG) | - | CoDA | | Right F2 | QSTTLKR | [GCA](http://bindr.gdcb.iastate.edu:8080/ZiFDB/controller/searchFinger?target=GCA) | - | CoDA | | Right F3 | RSDHLSL | [TGG](http://bindr.gdcb.iastate.edu:8080/ZiFDB/controller/searchFinger?target=TGG) | - | CoDA |   [ZF DNA Sequence](javascript:CoDAPopupNucleaseWindow("Left-ZFN-unknown-SP-6-1","RTDRLIR","QSAHLKR","RNTALQH","Right-ZFN-unknown-SP-6-1","RSQTLAQ","QSTTLKR","RSDHLSL")) | |
| --- | --- | --- | --- | --- | --- | --- | --- | --- | --- | --- | --- | --- | --- | --- | --- | --- | --- | --- | --- | --- | --- | --- | --- | --- | --- | --- | --- | --- | --- | --- | --- | --- | --- | --- | --- | --- | --- | --- | --- |
| [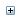](javascript:TreeView_ToggleNode(ctl00_ContentPlaceHolder1_tree12_Data,6,document.getElementById('ctl00_ContentPlaceHolder1_tree12n6'),'%20',document.getElementById('ctl00_ContentPlaceHolder1_tree12n6Nodes'))) | | ZFN-unknown-SP-7-2 1413 aGGCTGCCTATCAGAAA[GTGGTGGCT](http://bindr.gdcb.iastate.edu:8080/ZiFDB/controller/searchArray?site=GCTGTGGTG)g 1439  1413 t[CCGACGGAT](http://bindr.gdcb.iastate.edu:8080/ZiFDB/controller/searchArray?site=GCCGCATAG)AGTCTTTCACCACCGAc 1439 | |  |
