# Supplementary material for: Zinc finger nucleases for targeted mutagenesis and repair of the sickle-cell disease mutation: An in-silico study
Source: BMC Blood Disord. 2012 May 14;12:5. doi: 10.1186/1471-2326-12-5 (PMC3407482; doi:10.1186/1471-2326-12-5)
Supplement: Additional file 4 — A detailed list of the 8,954 bp located to the 5′ end of theβAchain gene. This file offers FASTA-format listing of the 8,954 bp located to the 5′ end of the βA chain gene. (NCBI reference sequence 5190000 to 5198951 is |NT_009237.18|) [file 1471-2326-12-5-S4.doc]

# Homo sapiens chromosome 11 genomic contig, GRCh37.p2 reference primary assembly

NCBI Reference Sequence: NT_009237.18

Showing 8.95kb region from base 5190000 to 5198951.

>gi|224514737:5190000-5198951 Homo sapiens chromosome 11 genomic contig, GRCh37.p2 reference primary assembly

GATTATAGAGGTAAGAGGGATAAAATTTAAGTATTTTCTTTTTATATTCATTCCTCTGTAAAAAACTAAA

GCAATGAGGATCTAGGCACACGTGTATCCCTGAGAAAAGATTTCACATGTTGAATCCTGGGAAAAGACGT

CTTTAAAATATTTTAAATGTTAAAACATGCAGATTTGACTTGGCTGTTAGATTTTGGATTTTATTTTATT

AAATTTAAACCTGCATTAGCATTGTTTTAGATTTAGACAGTTTTCAAGACCCTGTTTCACATCCCTGATA

TAAGAGGCATGTATATGTGAAATAAAGTGTTCTGCGGAAGTTTGAATATGTCTTTTGCAAATATCCTGGG

TCAAAGAAAATGCACAGACTTTATGAAATTATAATATAGGTTATATTTATAGTATTCTGAAAGACCAAAA

TTGTCAAAGCAAGAGCTTTGAAATCCTGATTGGCAGAAATTGTCACCTTCTTAAAGACTTACCCTACAAC

TTCTTATGCTCAGAAATAGTTTTCCTTTTTCTATTTTGTTTTCTTTTTTAAATGATGAACTATGGATCCT

TCTCTTGTGTTGGCAACTGCTGCAGATACCATCATCCTGGCTTCAAGGCAGGGGTTGCTTTTCCAATGGT

AGTTACTTAGTGTGACTAGAGTGTAACGCAGACTTTTCTTTCTATTACCCATAATACCCTGCAGGGACAA

GGCTGCAAGCTATACTAAGACCATCAAAAGCCCAGGCATACCAGGCAAATAAGTTTCAAGAAGCAATAAA

TAGTGCAAATTTGGTTATGGTCAGAGCCTCAGTTTCAAATCTAAATCAGCATTCAAAGTTCCTGAAAAAC

TATTCAAGCTTACTGACATCTTCACTATTGTGAGCTTGCTTCTACTCTGTGAATGGATGCCACAGCAGGT

GCAGGTCTATTCTACTTTTATTCCAGCCCCACTGACCACAACACACACACACACACACACACACACACAC

ACACAAGTCCTCAAAAAAGAGACAGCGAGAGAGACAGAGGTCCATTTCCTTTACTCCAGTCTATTAATCT

TTCTTAAGTCCTAATGTTTTATATCAGAATGGCCCTAGTCTGGGTATACTTAGAGGATGAATATTAACTT

CCTCCATGAAAATCATGACACTCATCTATGGGAGTGTGGTTTTATCATTTGTTTCAAAAGCAGCACTTGA

CTAGAGTATTTTTATACATGCTCTACTGTTTAGTCTAAAATTCCCCAAGTGAGACATTTTAGCAATCTAC

TGTATATGTTCCTAGGTCAAGCACAGTCTAGCTACTTGCTACAGACTTCTCTCGCAGATACACAATCTCT

CAGTAAACACAACTCAGCAGCTCTCAGAATTAATTAATTAAAATGAAATAAAAATGCTCCAAGAAAACCA

GAACCCATAGAAACAAACCGCACACACACAAAATAGTTTGGAAATAGGATAGTTTAGTTCACTTTAAAAA

GTTGTATTTCTTACCAAAAAGCAATAAAGTCAACATAGACAGAACTTTGCTAAAGTATATGCATAATTAT

TACAGAAAAATTTATATTTCAGTCTGCACCTTGTTCATTAATATTTCCTCCACTGGGCTCAGATTTTCAT

TCAGAATTAGCTTTTTTTTAACCTCATTAAGTGGGCCATGGTGTCAATTTATGGAGAGCAGAGGTACAGT

CTTCAGATAAATTTGAGATTGCATCTCTTTAGAGGTAGAATCTTGGCTTCAGTCTGAACACCCTTTACAA

ACATGTAGGCATTAATTCAGAGGACTGCTCTGAAGCTCAAGAGATGGATGATTTAGGAGACTATTAGGCT

TAATTCTCCTGACATTAAATTATTTTATTGAAAAATTTTCATGAACTCAAATTCACATTATTACGTCCTC

TCTTCCCTTCCTCTCTCCCTCTCTTTCACACACACACACACCCTTTCATTCAGACATACTGAACATAGTT

TATAAAGCAACGCCATAGTGAGAAAAGAAAAACAACCATTTGATAAATTATCAAATAAAATTAAAGCCAA

ATCTTGAGGAATTAATTCCATTCCTATACTTTGTTTTAACTTTCATTTTAAATTTGGGGGTACATGTACA

AGTTATATAGGTAAATTGCATATCACTGGGTCTTGATGTACAGATTATTTCATCACTCAGTGATGAACTT

AATACCCAATAGGTTCTTTTTCATCGTCTCCCTCTTCCCACCCTCAACCCTCAAGTAGGCCTGGGTGTCT

GTTGATCTATTTTTTGTGTCCATGTGTATCATCATTTAGCTCCCACACTCCTAGACTCTTACAAAAGCTC

TACTCTAACAAATAATTTTAATATAACAACAAGGTGCCAAGTCTTTTTTCATCTCTTGACCTCCTCATCT

TCAATATGAAGATAGCAATGCCTAGCATATAGTAATTGTGTGTGCTCGGCACATGTCCCATCCAGGTGAT

GTTCTCATGAATTCTCTGGTATTTGGATTTTTAGGGCCAACATCTTGCCTAGACTCACTGACTACTTCTA

ATGGTGTAGGAATAGAAGGTATAGTAAGCTATATTTGCTCTTTTTTTTTGACTTTTGGAGAAAGGAATGT

AGTTTGTTTTCCCTATCAAGATCATAAGGTCTACAATCTCTCTTACTGTCTTTCGAATACCACACAAGTA

TCTCCTTTTAATATCTTACTCTTATGAGAACTTCCCTATGCTCAGGAATGTGAAAGTGATCTTCCCCAAA

ATGCCCTGAGTAGCTCTGCTTCTGATATGTAAACCAGTGGTTCCTAAATATCTTATTCAGATATAAAGAG

TTCTCAGTGCCTACTATGTATAATGACTTAAGGAAAACACATGGATATAAAAGAGCTTCTGCTGCCAGTC

AATTCAAAATGTTGTGATCAAAACAGTTAACTATAACACATTGTCAGTATATTCTGGCAGTACAGACAGA

GTTACTGTGAAAGCACTTTCAAACACAAGGAAACAACAGATTCTGTTTAGGTATTCAAGTAAGTTTAAAA

TTAATCAACATGCATATAATACAGATTTTTAAAAATGTATACAACTGAGAAAGTTCTAAAAATCCTTCTA

ATTTTACCTATGGCAAAAATGGTGCTCAAGGAAAAAAATATTACTCCTATGATCAGAAAGGAAATAACTG

TTCGGTTGTTTACTGCATGCTTCCTCTTTGTATGTTGTAGCATTAACAACAAAGAAACACTGTGCGATGG

TATTATTAATATTGTCCTCTAATTCCAGAAGTTCTAAAGCAAGGAATAATCATTCCTTCATTTACTCATT

TACTCATTCGTTCATTCATTCACTGTTTTCTTGAATAAATATTTTCTTTCTGCCCATTGATGAATTTAAT

ATGAGGCACAGTAGGCATGTAAGAAGGTAGAGAATAGGCAGAATTGCTTATAAAGCACGGAGTGTGTGTG

TGTGCATGAAATAAATAAGAAAAATATAAAAATATAAAAAATGGTGTGGGGGAGGGTTTGGAAAGATTTT

TTGGGTAGTATAGAGAAGTTTATTGTGGCTGGGGTCAAGAGAGGTCACAAGTAATACGTGAGCAATGAAT

CTTGACTGAAATATGGGAAGATAAGAGGAAATTCTTTACAGAGATGTTCTGGGGCAAGTAAGAGGAGGAA

GCTATTTCTTGGAGCAGGAACACTTGATGGGGTATAGTATTATGGGCTACAATGTGCAGGCAAAGGAAGG

AGGAAGAAAGACAACATAACATTGTCTTGAGTAATCATTATGCCTTTAATTGAGCACATTATTTTCTCAG

TAATTGTTGGAGTTTAATCGTAGCATTACCCTTGAGGCTATGTCTTAGGGTTGAGGTCTTCCCTAGAACC

TCTGCAGTGCCAGTATTATCTTTGTATCAAGAGTTCTTGATAATTTCTGCTCTTTGGAGGTAGAAGTGTC

ACCCATTAATGCCTTGTACGGTTCCCTTGCTTTTCTCTTTTCCCATGTACTCTTTGTAAAATAAACAAGT

GCTCCCTATCTGTAGAGCCTCAGGAACCTTCTTACACACCTGGACAAAAAAATGAAATAAGTGAAATTAA

TCAGGAAGTTGAGCTGAACATTCTTTATTAGGCAGAAGCCATACCCTTGAAGTAGGCATTGTGTTCCCAA

GTTCAGAAAATAGAATCTAGGGAAATAGGGTCTTCTTATGGTTATCAGGAAACAGTCCAGGATCTCAATG

GTACTTGTGAGCCAGGGCATTAGCCACACCAGCCACCACCTTCTGATAGGCAGCCTGCATTTGTGGGGTG

AATTCCTTGCCAAAGTTGCGGGCCAGCACACACACCAGCACATTGCCCAAGAGCTGCGGAGAAGAGGTAG

GCAGATACATGCATATGGTTAACAGAGAAATAAAGACTGGCTTCTGAGAAACTGAGCCAACACCCATTTT

TTTCTGCCCAAATCTTAGACAAAACTGATCCCCAGGTTATTCCCATCAGCATAAATAAGTACATATATGA

ATGCATACATATAACATATATCTATACACACACATCCTCTATGTACTTAACTAGCATGTAGTCTATATAT

GTACATATATGCTATATATGCTGTATAATACTATATACAAATTAATTCCAAATTAGTTTTAATTTTGTAT

GTGTATATAGCATATACAAATTAATTACTATAAACAGATTAATAGATACAAATTAATTGATTAATCAGTG

TGATGATGGGCTGTCTCCTAGCAACGACTTCTGCCCCACCTCCAGTGTAACTGCCTAGTCTTTCATAATC

AAATATTCACTTTCCTTTCCATTCCATTTACTACAGAATTTATAAAATTCCAATTATTCCTTATTGTAAA

ATGATTTATAGCCTCTAAAACAGTATTCTATGCCTCTCATCTTTGAGTTGGAGCCTCTCCCATACCCATG

TGGAGAGACAAAAGGATTATTCTAAGTGCAGAATTAGCAGGTGAGAGCTGGTATGCATAATTTGAGTTGT

TGTTAGAGAAGGAAAAATGAAGGGAGGGGGTTGGGAGAGAAAGACAGGATATTAAATAATTTAAAATAGC

AAGATTGTGAGGAAGGAAAAAATGCAGAATATTTAAATAAAAAATTAACAAAATTTTAGAAGCATTAAAT

GATAAAATATAGTAAAATGACAAAAATGTGGGAGAAGAGCAGGTAGGTAAAAGAACCAAAATGTAAGATT

AGAAAGTAAAAAGAGAAAAGTGAAGCATCTCCTGGACTCACCCTGAAGTTCTCAGGATCCACGTGCAGCT

TGTCACAGTGCAGCTCACTCAGCTGAGAAAAAGTGCCCTTGAGGTTGTCCAGGTGAGCCAGGCCATCACT

AAAGGCACCTAGCACCTTCTTGCCATGAGCCTTCACCTTAGGGTTGCCCATAACAGCATCAGGAGAGGAC

AGATCCCCAAAGGACTCAAAGAACCTCTGGGTCCAAGGGTAGACCACCAGTAATCTGAGGGTAGGAAAAC

AGCCCAAGGGACAGAGAGTCAGTGCCTATCAGAAACCCAAGAGTCTTCTCTGTCTACACATGCCCAGTTT

CCATTTGCCTCCTTGAGCCTCTCTTATAACCTTGATACCAACCTGCCCAGGGCCTCACCACCAACTGCAT

CCACGTTCACTTTGCCCCACAGGGCATTGACAGCAGTCTTCTCCTCAGGAGTCAGATGCACCATGGTGTC

TGTTTGAGGTTGCTAGTGAACACTGTTATGTCAGAAGAAAGTGTAAGCAACAGTCGACTCTGCCCTGCCT

TTTATGCTGGTCCTGTCCTCCCTGCTCCAGTGAGCAGGTTGGTTTAAGATAAGCAGGGTTTCATTAGTTT

GTGAGAATGAAAAATGAACCTTCATTCCACTATTCCCTTAACTTGCCCTGAGATTGGCTGTTCTGTCATG

TGTGTCTTGACTCAGAAACCCTGTTCTCCTCTACATATCTCCCCACCGCATCTCTTTCAGCAGTTGTTTC

TAAAAATATCCTCCTAGTTTCATTTTTGCAGAAGTGTTTTAGGCTAATATAGTGGAATGTATCTTAGAGT

TTAACTTATTTGTTTCTGTCACTTTATACTAAGAAAACTTATCTAAAAGCAGATGTTTTAACAAGTTGAC

TCAATATAAAGTTCTTCTTTGCCTCTAGAGATTTTTGTCTCCAAGGGAATTTTGAGAGGTTGGAATGGAC

AAATCTATTGCTGCAGTTTAAACTTGCTTGCTTCCTCCTTCTTTTGGTAAATTCTTCCTATAATAAAACT

CTAATTTTTTATTATATTGAAATAAATATCCATTAAAAGAATATTTAAAAAATGAATAGTGTTTATTTAC

CAGTTATTGAAATAGGTTCTGGAAACATGAATTTTAAGGTTAACATTTTAATGACAGATAAAATCAAATA

TTATATACAAATATTTTGAATGTTTAAAATTATGGTATGACTAAAGAAAGAATGCAAAGTGAAAAGTAGA

TTTACCATATTCAGCCAGATTAAATTTAACGAAGTTCCTGGGAATATGCTAGTACAGAACATTTTTACAG

ATGTGTTCTTAAAAAAAAATGTGGAATTAGACCCAGGAATGAAGATCCCAGTAGTTTTTCACTCTTTTCT

GAATTCAAATAATGCCACAATGGCAGACAAATACACACCCATGAGCATATCCAAAAGGAAGGATTGAAGG

AAAGAGGAGGAAGAAATGGAGAAAGGAAGGAAGGAAGAGGGGAAGAGAGAGGATGGAAGGGATGGAGGAG

AAGAAGGAAAAATAAATAATGGAGAGGAGAGGAGAAAAAAGGAGGGGAGAGGAGAGGAGAAGGGATAGGG

AAGAGAAAGAGAAAGGGAAGGGAAGAGAGGAAAGAAGAGAAGAGGAGAGAAAAGAAACGAAGAGAGGGGA

AGGGAAGGAAAAAAAAGAGGAAAAAAGAGACAAGAGAAGAGATAAGACTGACAGTTCAAATTTTGGTGGT

GATATGGATCAATAGAAACTCAAACTCTGTTGGTGACACTGTACAATAGTATAACCCCTTTGGAAAACCT

TTAATAGTATCCACAAATGCTGGATGCTTGATAAGTCTATTACCTAGCAATTACATTTTTAGATATTCAG

AAACACATGCATGTGTGTATCCAAAGACATGTATAGAAATGCTTATGACAGCAATAATCATAAAAACCTC

AAACCGGTAGCCACTTAAATGCTTACCAACAGTAGAATTGATAAATTACGGTATAGTCAAAGAATAGAAT

ATTACACAGAAATGAAAAGAATCAACTACTGCTTAACACGTAGCGATACAAATGCATTTTACAGCATTTG

GTTGATTAAAAGTAACCAGAGGTGAGTTCAAACTATATGACTTTATTTGTATATAGAAAGATGGATGATG

TGCCTGAGATTCTGATCACAAGGGGAAATGTTATAAAATAGGGTAGAGAGGAGCCATGAATGACCTTTAA

ACTTTGTTACAAGTTATTTTTCTGTAACCTGGAAGCCAACGAAAGATATTGAATAATTCAAGAAAGGTGG

TGGCATGGTTTGATTTGTGTCTTTAAAAGATTATTCTCACTTAGTGAAGAAATGTATTTTAGAAGTAGAG

AAAATGGGAGACAAATAGCTGGGCTTCTGTTGCAGTAGGGAAGAAAGTGACAATGCCATTTCTATTATCA

GACTTGGACCATGACGGTGATGTCAGTCGTGAACACAAGAATAGGGCCACATTTGTGAGTTTAGTGGTAC

GATAAAATCAGAAATACAGTCTTGGATACATTGTATTGTATGCACTCTTGTAAAATGCAAAAAGATGTAC

TTAGATATGTGGATCTGGAGCTCAGAAAGAATACAACCAGGTCAAGAATACAGAATGGAACAGAACATAC

AAGAACAGATCATAATGTGCTGTGTGAATCACTACCACTACCTGTTAAAAATGACAGATGATGTACTTCA

TCAATATCTCCTTAAAATCTTAGAATGTGTTTGTGAGGGAGGAATTATGTTTCCAATTCATATATAAGAA

AATTGATTCTAAAAAAAATGTTAGGTAAATTCTTAAGGCCATGAGGACTGTTATTTGATCTTTGTCTGTT

AATTCCAAAGACTTGGCTTTTCACTTTAATTCTGTTCTACCTGAAATGATTTTACACATTGGGAGATCTG

GTTACATGTTTATTCTATATGGATTGCATTGAGAGGATTTGTATAACAGAATAAGGTCTTTTTTTCTTTT

CTCTTCTGAGATGGAGTTTCATCCCTATTGCCCAAGCTAGAGTGCAATGGTGCAATCTAGGCTCACCGCA

ACCTCTGCCTCCTGGGTTCAAGCAATTCTCCTGCCTCAGCCACCTGAATAGCTGGGACTGCAGGCATGCA

CCACACGCCCGGCTGATTTTGTATTTTTAGTAGAGATGGGGTTTCACCATGTTGGTCAGGCTGGTCTTGA

ACTCCTGACCTCAAGTGATCTGCCTGCCTTGGCCTCCCAAAGTGCTGGGTTTACAAGCCTGAGCCACCGC

ATCCAGCCAGGATAAGGTCTAAAAGTGGAAAGAATAGCATCTACTCTTGTTCAGGAAACAATGAGGACCT

GACTGGGCAGTAAGAGTGGTGATTAATAGATAGGGACAAATTGAAGCAGAATCGAACTGTTGATTAGAGG

TAGGGAAATGATTTTAATCTGTGACCTTGGTGAATGGGCAAGTAGCTATCTAATGACTAAAATGGAAAAC

ACTGGAAGAGAAACAGTTTTAGTATAACAAGTGAAATACCCATGCTGAGTCTGAGGTGCCTATAGGACAT

CTATATAAATAAGCCCAGTACATTGTTTGATATATGGGTTTGGCACTGAGGTTGGAGGTCAGAGGTTAGA

AATCAGAGTTGGGAATTGGGATTATACAGGCTGTATTTAAGAGTTTAGATATAACTGTGAATCCAAGAGT

GTGATGAATACAAAGTTAAATGAAGGACCTTTAATGAACACCAACATTTAATGTGAAATCTC
