# Supplementary material for: Zinc finger nucleases for targeted mutagenesis and repair of the sickle-cell disease mutation: An in-silico study
Source: BMC Blood Disord. 2012 May 14;12:5. doi: 10.1186/1471-2326-12-5 (PMC3407482; doi:10.1186/1471-2326-12-5)
Supplement: Additional file 5 — A detailed list of thefiveZFNs specific to the 8,954 bp located to the 5′ end of theβAchain gene. This file lists the fives ZFNs that specifically bind and cleave sequences within the 8,954 bp located to the 5′ end of the βA chain gene. [file 1471-2326-12-5-S5.doc]

**Zinc Finger Site Type:** Nuclease
**Zinc Finger Engineering Method:** CoDA
**Sequence Name** : unknown
**Sequence Length**:8954
**Nucleotide Sequence** :nGATTATAGAGGTAAGAGGGATAAAATTTAAGTATTTTCTTTTTATATTCATTCCTCTGTAAAAAACTAAAGCAATGAGGATCTAGGCACACGTGTATCCCTGAGAAAAGATTTCACATGTTGAATCCTGGGAAAAGACGTCTTTAAAATATTTTAAATGTTAAAACATGCAGATTTGACTTGGCTGTTAGATTTTGGATTTTATTTTATTAAATTTAAACCTGCATTAGCATTGTTTTAGATTTAGACAGTTTTCAAGACCCTGTTTCACATCCCTGATATAAGAGGCATGTATATGTGAAATAAAGTGTTCTGCGGAAGTTTGAATATGTCTTTTGCAAATATCCTGGGTCAAAGAAAATGCACAGACTTTATGAAATTATAATATAGGTTATATTTATAGTATTCTGAAAGACCAAAATTGTCAAAGCAAGAGCTTTGAAATCCTGATTGGCAGAAATTGTCACCTTCTTAAAGACTTACCCTACAACTTCTTATGCTCAGAAATAGTTTTCCTTTTTCTATTTTGTTTTCTTTTTTAAATGATGAACTATGGATCCTTCTCTTGTGTTGGCAACTGCTGCAGATACCATCATCCTGGCTTCAAGGCAGGGGTTGCTTTTCCAATGGTAGTTACTTAGTGTGACTAGAGTGTAACGCAGACTTTTCTTTCTATTACCCATAATACCCTGCAGGGACAAGGCTGCAAGCTATACTAAGACCATCAAAAGCCCAGGCATACCAGGCAAATAAGTTTCAAGAAGCAATAAATAGTGCAAATTTGGTTATGGTCAGAGCCTCAGTTTCAAATCTAAATCAGCATTCAAAGTTCCTGAAAAACTATTCAAGCTTACTGACATCTTCACTATTGTGAGCTTGCTTCTACTCTGTGAATGGATGCCACAGCAGGTGCAGGTCTATTCTACTTTTATTCCAGCCCCACTGACCACAACACACACACACACACACACACACACACACACACAAGTCCTCAAAAAAGAGACAGCGAGAGAGACAGAGGTCCATTTCCTTTACTCCAGTCTATTAATCTTTCTTAAGTCCTAATGTTTTATATCAGAATGGCCCTAGTCTGGGTATACTTAGAGGATGAATATTAACTTCCTCCATGAAAATCATGACACTCATCTATGGGAGTGTGGTTTTATCATTTGTTTCAAAAGCAGCACTTGACTAGAGTATTTTTATACATGCTCTACTGTTTAGTCTAAAATTCCCCAAGTGAGACATTTTAGCAATCTACTGTATATGTTCCTAGGTCAAGCACAGTCTAGCTACTTGCTACAGACTTCTCTCGCAGATACACAATCTCTCAGTAAACACAACTCAGCAGCTCTCAGAATTAATTAATTAAAATGAAATAAAAATGCTCCAAGAAAACCAGAACCCATAGAAACAAACCGCACACACACAAAATAGTTTGGAAATAGGATAGTTTAGTTCACTTTAAAAAGTTGTATTTCTTACCAAAAAGCAATAAAGTCAACATAGACAGAACTTTGCTAAAGTATATGCATAATTATTACAGAAAAATTTATATTTCAGTCTGCACCTTGTTCATTAATATTTCCTCCACTGGGCTCAGATTTTCATTCAGAATTAGCTTTTTTTTAACCTCATTAAGTGGGCCATGGTGTCAATTTATGGAGAGCAGAGGTACAGTCTTCAGATAAATTTGAGATTGCATCTCTTTAGAGGTAGAATCTTGGCTTCAGTCTGAACACCCTTTACAAACATGTAGGCATTAATTCAGAGGACTGCTCTGAAGCTCAAGAGATGGATGATTTAGGAGACTATTAGGCTTAATTCTCCTGACATTAAATTATTTTATTGAAAAATTTTCATGAACTCAAATTCACATTATTACGTCCTCTCTTCCCTTCCTCTCTCCCTCTCTTTCACACACACACACACCCTTTCATTCAGACATACTGAACATAGTTTATAAAGCAACGCCATAGTGAGAAAAGAAAAACAACCATTTGATAAATTATCAAATAAAATTAAAGCCAAATCTTGAGGAATTAATTCCATTCCTATACTTTGTTTTAACTTTCATTTTAAATTTGGGGGTACATGTACAAGTTATATAGGTAAATTGCATATCACTGGGTCTTGATGTACAGATTATTTCATCACTCAGTGATGAACTTAATACCCAATAGGTTCTTTTTCATCGTCTCCCTCTTCCCACCCTCAACCCTCAAGTAGGCCTGGGTGTCTGTTGATCTATTTTTTGTGTCCATGTGTATCATCATTTAGCTCCCACACTCCTAGACTCTTACAAAAGCTCTACTCTAACAAATAATTTTAATATAACAACAAGGTGCCAAGTCTTTTTTCATCTCTTGACCTCCTCATCTTCAATATGAAGATAGCAATGCCTAGCATATAGTAATTGTGTGTGCTCGGCACATGTCCCATCCAGGTGATGTTCTCATGAATTCTCTGGTATTTGGATTTTTAGGGCCAACATCTTGCCTAGACTCACTGACTACTTCTAATGGTGTAGGAATAGAAGGTATAGTAAGCTATATTTGCTCTTTTTTTTTGACTTTTGGAGAAAGGAATGTAGTTTGTTTTCCCTATCAAGATCATAAGGTCTACAATCTCTCTTACTGTCTTTCGAATACCACACAAGTATCTCCTTTTAATATCTTACTCTTATGAGAACTTCCCTATGCTCAGGAATGTGAAAGTGATCTTCCCCAAAATGCCCTGAGTAGCTCTGCTTCTGATATGTAAACCAGTGGTTCCTAAATATCTTATTCAGATATAAAGAGTTCTCAGTGCCTACTATGTATAATGACTTAAGGAAAACACATGGATATAAAAGAGCTTCTGCTGCCAGTCAATTCAAAATGTTGTGATCAAAACAGTTAACTATAACACATTGTCAGTATATTCTGGCAGTACAGACAGAGTTACTGTGAAAGCACTTTCAAACACAAGGAAACAACAGATTCTGTTTAGGTATTCAAGTAAGTTTAAAATTAATCAACATGCATATAATACAGATTTTTAAAAATGTATACAACTGAGAAAGTTCTAAAAATCCTTCTAATTTTACCTATGGCAAAAATGGTGCTCAAGGAAAAAAATATTACTCCTATGATCAGAAAGGAAATAACTGTTCGGTTGTTTACTGCATGCTTCCTCTTTGTATGTTGTAGCATTAACAACAAAGAAACACTGTGCGATGGTATTATTAATATTGTCCTCTAATTCCAGAAGTTCTAAAGCAAGGAATAATCATTCCTTCATTTACTCATTTACTCATTCGTTCATTCATTCACTGTTTTCTTGAATAAATATTTTCTTTCTGCCCATTGATGAATTTAATATGAGGCACAGTAGGCATGTAAGAAGGTAGAGAATAGGCAGAATTGCTTATAAAGCACGGAGTGTGTGTGTGTGCATGAAATAAATAAGAAAAATATAAAAATATAAAAAATGGTGTGGGGGAGGGTTTGGAAAGATTTTTTGGGTAGTATAGAGAAGTTTATTGTGGCTGGGGTCAAGAGAGGTCACAAGTAATACGTGAGCAATGAATCTTGACTGAAATATGGGAAGATAAGAGGAAATTCTTTACAGAGATGTTCTGGGGCAAGTAAGAGGAGGAAGCTATTTCTTGGAGCAGGAACACTTGATGGGGTATAGTATTATGGGCTACAATGTGCAGGCAAAGGAAGGAGGAAGAAAGACAACATAACATTGTCTTGAGTAATCATTATGCCTTTAATTGAGCACATTATTTTCTCAGTAATTGTTGGAGTTTAATCGTAGCATTACCCTTGAGGCTATGTCTTAGGGTTGAGGTCTTCCCTAGAACCTCTGCAGTGCCAGTATTATCTTTGTATCAAGAGTTCTTGATAATTTCTGCTCTTTGGAGGTAGAAGTGTCACCCATTAATGCCTTGTACGGTTCCCTTGCTTTTCTCTTTTCCCATGTACTCTTTGTAAAATAAACAAGTGCTCCCTATCTGTAGAGCCTCAGGAACCTTCTTACACACCTGGACAAAAAAATGAAATAAGTGAAATTAATCAGGAAGTTGAGCTGAACATTCTTTATTAGGCAGAAGCCATACCCTTGAAGTAGGCATTGTGTTCCCAAGTTCAGAAAATAGAATCTAGGGAAATAGGGTCTTCTTATGGTTATCAGGAAACAGTCCAGGATCTCAATGGTACTTGTGAGCCAGGGCATTAGCCACACCAGCCACCACCTTCTGATAGGCAGCCTGCATTTGTGGGGTGAATTCCTTGCCAAAGTTGCGGGCCAGCACACACACCAGCACATTGCCCAAGAGCTGCGGAGAAGAGGTAGGCAGATACATGCATATGGTTAACAGAGAAATAAAGACTGGCTTCTGAGAAACTGAGCCAACACCCATTTTTTTCTGCCCAAATCTTAGACAAAACTGATCCCCAGGTTATTCCCATCAGCATAAATAAGTACATATATGAATGCATACATATAACATATATCTATACACACACATCCTCTATGTACTTAACTAGCATGTAGTCTATATATGTACATATATGCTATATATGCTGTATAATACTATATACAAATTAATTCCAAATTAGTTTTAATTTTGTATGTGTATATAGCATATACAAATTAATTACTATAAACAGATTAATAGATACAAATTAATTGATTAATCAGTGTGATGATGGGCTGTCTCCTAGCAACGACTTCTGCCCCACCTCCAGTGTAACTGCCTAGTCTTTCATAATCAAATATTCACTTTCCTTTCCATTCCATTTACTACAGAATTTATAAAATTCCAATTATTCCTTATTGTAAAATGATTTATAGCCTCTAAAACAGTATTCTATGCCTCTCATCTTTGAGTTGGAGCCTCTCCCATACCCATGTGGAGAGACAAAAGGATTATTCTAAGTGCAGAATTAGCAGGTGAGAGCTGGTATGCATAATTTGAGTTGTTGTTAGAGAAGGAAAAATGAAGGGAGGGGGTTGGGAGAGAAAGACAGGATATTAAATAATTTAAAATAGCAAGATTGTGAGGAAGGAAAAAATGCAGAATATTTAAATAAAAAATTAACAAAATTTTAGAAGCATTAAATGATAAAATATAGTAAAATGACAAAAATGTGGGAGAAGAGCAGGTAGGTAAAAGAACCAAAATGTAAGATTAGAAAGTAAAAAGAGAAAAGTGAAGCATCTCCTGGACTCACCCTGAAGTTCTCAGGATCCACGTGCAGCTTGTCACAGTGCAGCTCACTCAGCTGAGAAAAAGTGCCCTTGAGGTTGTCCAGGTGAGCCAGGCCATCACTAAAGGCACCTAGCACCTTCTTGCCATGAGCCTTCACCTTAGGGTTGCCCATAACAGCATCAGGAGAGGACAGATCCCCAAAGGACTCAAAGAACCTCTGGGTCCAAGGGTAGACCACCAGTAATCTGAGGGTAGGAAAACAGCCCAAGGGACAGAGAGTCAGTGCCTATCAGAAACCCAAGAGTCTTCTCTGTCTACACATGCCCAGTTTCCATTTGCCTCCTTGAGCCTCTCTTATAACCTTGATACCAACCTGCCCAGGGCCTCACCACCAACTGCATCCACGTTCACTTTGCCCCACAGGGCATTGACAGCAGTCTTCTCCTCAGGAGTCAGATGCACCATGGTGTCTGTTTGAGGTTGCTAGTGAACACTGTTATGTCAGAAGAAAGTGTAAGCAACAGTCGACTCTGCCCTGCCTTTTATGCTGGTCCTGTCCTCCCTGCTCCAGTGAGCAGGTTGGTTTAAGATAAGCAGGGTTTCATTAGTTTGTGAGAATGAAAAATGAACCTTCATTCCACTATTCCCTTAACTTGCCCTGAGATTGGCTGTTCTGTCATGTGTGTCTTGACTCAGAAACCCTGTTCTCCTCTACATATCTCCCCACCGCATCTCTTTCAGCAGTTGTTTCTAAAAATATCCTCCTAGTTTCATTTTTGCAGAAGTGTTTTAGGCTAATATAGTGGAATGTATCTTAGAGTTTAACTTATTTGTTTCTGTCACTTTATACTAAGAAAACTTATCTAAAAGCAGATGTTTTAACAAGTTGACTCAATATAAAGTTCTTCTTTGCCTCTAGAGATTTTTGTCTCCAAGGGAATTTTGAGAGGTTGGAATGGACAAATCTATTGCTGCAGTTTAAACTTGCTTGCTTCCTCCTTCTTTTGGTAAATTCTTCCTATAATAAAACTCTAATTTTTTATTATATTGAAATAAATATCCATTAAAAGAATATTTAAAAAATGAATAGTGTTTATTTACCAGTTATTGAAATAGGTTCTGGAAACATGAATTTTAAGGTTAACATTTTAATGACAGATAAAATCAAATATTATATACAAATATTTTGAATGTTTAAAATTATGGTATGACTAAAGAAAGAATGCAAAGTGAAAAGTAGATTTACCATATTCAGCCAGATTAAATTTAACGAAGTTCCTGGGAATATGCTAGTACAGAACATTTTTACAGATGTGTTCTTAAAAAAAAATGTGGAATTAGACCCAGGAATGAAGATCCCAGTAGTTTTTCACTCTTTTCTGAATTCAAATAATGCCACAATGGCAGACAAATACACACCCATGAGCATATCCAAAAGGAAGGATTGAAGGAAAGAGGAGGAAGAAATGGAGAAAGGAAGGAAGGAAGAGGGGAAGAGAGAGGATGGAAGGGATGGAGGAGAAGAAGGAAAAATAAATAATGGAGAGGAGAGGAGAAAAAAGGAGGGGAGAGGAGAGGAGAAGGGATAGGGAAGAGAAAGAGAAAGGGAAGGGAAGAGAGGAAAGAAGAGAAGAGGAGAGAAAAGAAACGAAGAGAGGGGAAGGGAAGGAAAAAAAAGAGGAAAAAAGAGACAAGAGAAGAGATAAGACTGACAGTTCAAATTTTGGTGGTGATATGGATCAATAGAAACTCAAACTCTGTTGGTGACACTGTACAATAGTATAACCCCTTTGGAAAACCTTTAATAGTATCCACAAATGCTGGATGCTTGATAAGTCTATTACCTAGCAATTACATTTTTAGATATTCAGAAACACATGCATGTGTGTATCCAAAGACATGTATAGAAATGCTTATGACAGCAATAATCATAAAAACCTCAAACCGGTAGCCACTTAAATGCTTACCAACAGTAGAATTGATAAATTACGGTATAGTCAAAGAATAGAATATTACACAGAAATGAAAAGAATCAACTACTGCTTAACACGTAGCGATACAAATGCATTTTACAGCATTTGGTTGATTAAAAGTAACCAGAGGTGAGTTCAAACTATATGACTTTATTTGTATATAGAAAGATGGATGATGTGCCTGAGATTCTGATCACAAGGGGAAATGTTATAAAATAGGGTAGAGAGGAGCCATGAATGACCTTTAAACTTTGTTACAAGTTATTTTTCTGTAACCTGGAAGCCAACGAAAGATATTGAATAATTCAAGAAAGGTGGTGGCATGGTTTGATTTGTGTCTTTAAAAGATTATTCTCACTTAGTGAAGAAATGTATTTTAGAAGTAGAGAAAATGGGAGACAAATAGCTGGGCTTCTGTTGCAGTAGGGAAGAAAGTGACAATGCCATTTCTATTATCAGACTTGGACCATGACGGTGATGTCAGTCGTGAACACAAGAATAGGGCCACATTTGTGAGTTTAGTGGTACGATAAAATCAGAAATACAGTCTTGGATACATTGTATTGTATGCACTCTTGTAAAATGCAAAAAGATGTACTTAGATATGTGGATCTGGAGCTCAGAAAGAATACAACCAGGTCAAGAATACAGAATGGAACAGAACATACAAGAACAGATCATAATGTGCTGTGTGAATCACTACCACTACCTGTTAAAAATGACAGATGATGTACTTCATCAATATCTCCTTAAAATCTTAGAATGTGTTTGTGAGGGAGGAATTATGTTTCCAATTCATATATAAGAAAATTGATTCTAAAAAAAATGTTAGGTAAATTCTTAAGGCCATGAGGACTGTTATTTGATCTTTGTCTGTTAATTCCAAAGACTTGGCTTTTCACTTTAATTCTGTTCTACCTGAAATGATTTTACACATTGGGAGATCTGGTTACATGTTTATTCTATATGGATTGCATTGAGAGGATTTGTATAACAGAATAAGGTCTTTTTTTCTTTTCTCTTCTGAGATGGAGTTTCATCCCTATTGCCCAAGCTAGAGTGCAATGGTGCAATCTAGGCTCACCGCAACCTCTGCCTCCTGGGTTCAAGCAATTCTCCTGCCTCAGCCACCTGAATAGCTGGGACTGCAGGCATGCACCACACGCCCGGCTGATTTTGTATTTTTAGTAGAGATGGGGTTTCACCATGTTGGTCAGGCTGGTCTTGAACTCCTGACCTCAAGTGATCTGCCTGCCTTGGCCTCCCAAAGTGCTGGGTTTACAAGCCTGAGCCACCGCATCCAGCCAGGATAAGGTCTAAAAGTGGAAAGAATAGCATCTACTCTTGTTCAGGAAACAATGAGGACCTGACTGGGCAGTAAGAGTGGTGATTAATAGATAGGGACAAATTGAAGCAGAATCGAACTGTTGATTAGAGGTAGGGAAATGATTTTAATCTGTGACCTTGGTGAATGGGCAAGTAGCTATCTAATGACTAAAATGGAAAACACTGGAAGAGAAACAGTTTTAGTATAACAAGTGAAATACCCATGCTGAGTCTGAGGTGCCTATAGGACATCTATATAAATAAGCCCAGTACATTGTTTGATATATGGGTTTGGCACTGAGGTTGGAGGTCAGAGGTTAGAAATCAGAGTTGGGAATTGGGATTATACAGGCTGTATTTAAGAGTTTAGATATAACTGTGAATCCAAGAGTGTGATGAATACAAAGTTAAATGAAGGACCTTTAATGAACACCAACATTTAATGTGAAATCTCn
**Selected Module Sets:**
**Left Module Count:** 3
**Spacer Nucleotide Count:** 5,6,7
**Right Module Count:** 3
**Ignore Asp Overlap:**False

The results below are zinc finger arrays that can be constructed using CoDA. Note that other methods (including modular assembly and OPEN) can also potentially be used to target the input sequence of interest.”

**Sort By: Hide intron splice sites**

**[

](http://zifit.partners.org/ZiFiT/CoDAZiFiTNuclease.aspx#ctl00_ContentPlaceHolder1_tree12_SkipLink)**

| [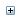](javascript:TreeView_ToggleNode(ctl00_ContentPlaceHolder1_tree12_Data,0,document.getElementById('ctl00_ContentPlaceHolder1_tree12n0'),'%20',document.getElementById('ctl00_ContentPlaceHolder1_tree12n0Nodes'))) | ZFN-unknown-SP-7-1 3167 aTGCTTCCTCTTTGTAT[GTTGTAGCA](http://bindr.gdcb.iastate.edu:8080/ZiFDB/controller/searchArray?site=GCAGTAGTT)t 3193  3167 t[ACGAAGGAG](http://bindr.gdcb.iastate.edu:8080/ZiFDB/controller/searchArray?site=GCAGAAGAG)AAACATACAACATCGTa 3193 |
| --- | --- |

|  | 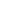 | | | FINGER | HELIX | TRIPLET | REFERENCE NUMBER | SOURCE | | --- | --- | --- | --- | --- | | Left F1 | RGQELRR | [GCA](http://bindr.gdcb.iastate.edu:8080/ZiFDB/controller/searchFinger?target=GCA) | - | CoDA | | Left F2 | QQTNLTR | [GAA](http://bindr.gdcb.iastate.edu:8080/ZiFDB/controller/searchFinger?target=GAA) | - | CoDA | | Left F3 | RRDNLNR | [GAG](http://bindr.gdcb.iastate.edu:8080/ZiFDB/controller/searchFinger?target=GAG) | - | CoDA | | Right F1 | HNGTLKR | [GCA](http://bindr.gdcb.iastate.edu:8080/ZiFDB/controller/searchFinger?target=GCA) | - | CoDA | | Right F2 | QRSSLVR | [GTA](http://bindr.gdcb.iastate.edu:8080/ZiFDB/controller/searchFinger?target=GTA) | - | CoDA | | Right F3 | HHNSLTR | [GTT](http://bindr.gdcb.iastate.edu:8080/ZiFDB/controller/searchFinger?target=GTT) | - | CoDA |   [ZF DNA Sequence](javascript:CoDAPopupNucleaseWindow("Left-ZFN-unknown-SP-7-1","RGQELRR","QQTNLTR","RRDNLNR","Right-ZFN-unknown-SP-7-1","HNGTLKR","QRSSLVR","HHNSLTR")) | |
| --- | --- | --- | --- | --- | --- | --- | --- | --- | --- | --- | --- | --- | --- | --- | --- | --- | --- | --- | --- | --- | --- | --- | --- | --- | --- | --- | --- | --- | --- | --- | --- | --- | --- | --- | --- | --- | --- | --- | --- |
| [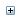](javascript:TreeView_ToggleNode(ctl00_ContentPlaceHolder1_tree12_Data,2,document.getElementById('ctl00_ContentPlaceHolder1_tree12n2'),'%20',document.getElementById('ctl00_ContentPlaceHolder1_tree12n2Nodes'))) | | ZFN-unknown-SP-7-2 4230 cAGCCACCACCTTCTGA[TAGGCAGCC](http://bindr.gdcb.iastate.edu:8080/ZiFDB/controller/searchArray?site=GCCGCATAG)t 4256  4230 g[TCGGTGGTG](http://bindr.gdcb.iastate.edu:8080/ZiFDB/controller/searchArray?site=GCTGTGGTG)GAAGACTATCCGTCGGa 4256 | |  |

|  | 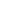 | | | FINGER | HELIX | TRIPLET | REFERENCE NUMBER | SOURCE | | --- | --- | --- | --- | --- | | Left F1 | TKPILVR | [GCT](http://bindr.gdcb.iastate.edu:8080/ZiFDB/controller/searchFinger?target=GCT) | - | CoDA | | Left F2 | RREVLEN | [GTG](http://bindr.gdcb.iastate.edu:8080/ZiFDB/controller/searchFinger?target=GTG) | - | CoDA | | Left F3 | RKDALHV | [GTG](http://bindr.gdcb.iastate.edu:8080/ZiFDB/controller/searchFinger?target=GTG) | - | CoDA | | Right F1 | DPSTLRR | [GCC](http://bindr.gdcb.iastate.edu:8080/ZiFDB/controller/searchFinger?target=GCC) | - | CoDA | | Right F2 | QSTTLKR | [GCA](http://bindr.gdcb.iastate.edu:8080/ZiFDB/controller/searchFinger?target=GCA) | - | CoDA | | Right F3 | RRDGLAG | [TAG](http://bindr.gdcb.iastate.edu:8080/ZiFDB/controller/searchFinger?target=TAG) | - | CoDA |   [ZF DNA Sequence](javascript:CoDAPopupNucleaseWindow("Left-ZFN-unknown-SP-7-2","TKPILVR","RREVLEN","RKDALHV","Right-ZFN-unknown-SP-7-2","DPSTLRR","QSTTLKR","RRDGLAG")) | |
| --- | --- | --- | --- | --- | --- | --- | --- | --- | --- | --- | --- | --- | --- | --- | --- | --- | --- | --- | --- | --- | --- | --- | --- | --- | --- | --- | --- | --- | --- | --- | --- | --- | --- | --- | --- | --- | --- | --- | --- |
| [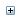](javascript:TreeView_ToggleNode(ctl00_ContentPlaceHolder1_tree12_Data,4,document.getElementById('ctl00_ContentPlaceHolder1_tree12n4'),'%20',document.getElementById('ctl00_ContentPlaceHolder1_tree12n4Nodes'))) | | ZFN-unknown-SP-6-1 4231 aGCCACCACCTTCTGA[TAGGCAGCC](http://bindr.gdcb.iastate.edu:8080/ZiFDB/controller/searchArray?site=GCCGCATAG)t 4256  4231 t[CGGTGGTGG](http://bindr.gdcb.iastate.edu:8080/ZiFDB/controller/searchArray?site=GGCGGTGGT)AAGACTATCCGTCGGa 4256 | |  |

|  | 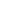 | | | FINGER | HELIX | TRIPLET | REFERENCE NUMBER | SOURCE | | --- | --- | --- | --- | --- | | Left F1 | VPSKLKR | [GGC](http://bindr.gdcb.iastate.edu:8080/ZiFDB/controller/searchFinger?target=GGC) | - | CoDA | | Left F2 | EAHHLSR | [GGT](http://bindr.gdcb.iastate.edu:8080/ZiFDB/controller/searchFinger?target=GGT) | - | CoDA | | Left F3 | IRHHLKR | [GGT](http://bindr.gdcb.iastate.edu:8080/ZiFDB/controller/searchFinger?target=GGT) | - | CoDA | | Right F1 | DPSTLRR | [GCC](http://bindr.gdcb.iastate.edu:8080/ZiFDB/controller/searchFinger?target=GCC) | - | CoDA | | Right F2 | QSTTLKR | [GCA](http://bindr.gdcb.iastate.edu:8080/ZiFDB/controller/searchFinger?target=GCA) | - | CoDA | | Right F3 | RRDGLAG | [TAG](http://bindr.gdcb.iastate.edu:8080/ZiFDB/controller/searchFinger?target=TAG) | - | CoDA |   [ZF DNA Sequence](javascript:CoDAPopupNucleaseWindow("Left-ZFN-unknown-SP-6-1","VPSKLKR","EAHHLSR","IRHHLKR","Right-ZFN-unknown-SP-6-1","DPSTLRR","QSTTLKR","RRDGLAG")) | |
| --- | --- | --- | --- | --- | --- | --- | --- | --- | --- | --- | --- | --- | --- | --- | --- | --- | --- | --- | --- | --- | --- | --- | --- | --- | --- | --- | --- | --- | --- | --- | --- | --- | --- | --- | --- | --- | --- | --- | --- |
| [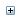](javascript:TreeView_ToggleNode(ctl00_ContentPlaceHolder1_tree12_Data,6,document.getElementById('ctl00_ContentPlaceHolder1_tree12n6'),'%20',document.getElementById('ctl00_ContentPlaceHolder1_tree12n6Nodes'))) | | ZFN-unknown-SP-6-2 4234 cACCACCTTCTGATAG[GCAGCCTGC](http://bindr.gdcb.iastate.edu:8080/ZiFDB/controller/searchArray?site=TGCGCCGCA)a 4259  4234 g[TGGTGGAAG](http://bindr.gdcb.iastate.edu:8080/ZiFDB/controller/searchArray?site=GGTGGTGAA)ACTATCCGTCGGACGt 4259 | |  |

|  | 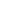 | | | FINGER | HELIX | TRIPLET | REFERENCE NUMBER | SOURCE | | --- | --- | --- | --- | --- | | Left F1 | MKHHLAR | [GGT](http://bindr.gdcb.iastate.edu:8080/ZiFDB/controller/searchFinger?target=GGT) | - | CoDA | | Left F2 | EAHHLSR | [GGT](http://bindr.gdcb.iastate.edu:8080/ZiFDB/controller/searchFinger?target=GGT) | - | CoDA | | Left F3 | QDGNLTR | [GAA](http://bindr.gdcb.iastate.edu:8080/ZiFDB/controller/searchFinger?target=GAA) | - | CoDA | | Right F1 | RGRNLEM | [TGC](http://bindr.gdcb.iastate.edu:8080/ZiFDB/controller/searchFinger?target=TGC) | - | CoDA | | Right F2 | DSSVLRR | [GCC](http://bindr.gdcb.iastate.edu:8080/ZiFDB/controller/searchFinger?target=GCC) | - | CoDA | | Right F3 | QGGTLRR | [GCA](http://bindr.gdcb.iastate.edu:8080/ZiFDB/controller/searchFinger?target=GCA) | - | CoDA |   [ZF DNA Sequence](javascript:CoDAPopupNucleaseWindow("Left-ZFN-unknown-SP-6-2","MKHHLAR","EAHHLSR","QDGNLTR","Right-ZFN-unknown-SP-6-2","RGRNLEM","DSSVLRR","QGGTLRR")) | |
| --- | --- | --- | --- | --- | --- | --- | --- | --- | --- | --- | --- | --- | --- | --- | --- | --- | --- | --- | --- | --- | --- | --- | --- | --- | --- | --- | --- | --- | --- | --- | --- | --- | --- | --- | --- | --- | --- | --- | --- |
| [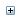](javascript:TreeView_ToggleNode(ctl00_ContentPlaceHolder1_tree12_Data,8,document.getElementById('ctl00_ContentPlaceHolder1_tree12n8'),'%20',document.getElementById('ctl00_ContentPlaceHolder1_tree12n8Nodes'))) | | ZFN-unknown-SP-7-3 5923 cCACCGCATCTCTTTCA[GCAGTTGTT](http://bindr.gdcb.iastate.edu:8080/ZiFDB/controller/searchArray?site=GTTGTTGCA)t 5949  5923 g[GTGGCGTAG](http://bindr.gdcb.iastate.edu:8080/ZiFDB/controller/searchArray?site=GTGGCGGAT)AGAAAGTCGTCAACAAa 5949 | |  |
